# Cytokine-induced molecular responses in airway smooth muscle cells inform genome-wide association studies of asthma

**DOI:** 10.1186/s13073-020-00759-w

**Published:** 2020-07-20

**Authors:** Emma E. Thompson, Quynh Dang, Blair Mitchell-Handley, Kavitha Rajendran, Sumati Ram-Mohan, Julian Solway, Carole Ober, Ramaswamy Krishnan

**Affiliations:** 1 Department of Human Genetics, The University of Chicago, Chicago, IL USA; 2 Center for Vascular Biology Research, Beth Israel Deaconess Medical Center, Harvard Medical School, Boston, MA USA; 3 Department of Medicine, The University of Chicago, Chicago, IL USA

**Keywords:** Asthma, Epigenetics, Differential expression, Cases, Controls

## Abstract

**Background:**

A challenge in the post-GWAS era is to assign function to disease-associated variants. However, available resources do not include all tissues or environmental exposures that are relevant to all diseases. For example, exaggerated bronchoconstriction of airway smooth muscle cells (ASMCs) defines airway hyperresponsiveness (AHR), a cardinal feature of asthma. However, the contribution of ASMC to genetic and genomic studies has largely been overlooked. Our study aimed to address the gap in data availability from a critical tissue in genomic studies of asthma.

**Methods:**

We developed a cell model of AHR to discover variants associated with transcriptional, epigenetic, and cellular responses to two AHR promoting cytokines, IL-13 and IL-17A, and performed a GWAS of bronchial responsiveness (BRI) in humans.

**Results:**

Our study revealed significant response differences between ASMCs from asthma cases and controls, including genes implicated in asthma susceptibility. We defined molecular quantitative trait loci (QTLs) for expression (eQTLs) and methylation (meQTLs), and cellular QTLs for contractility (coQTLs) and performed a GWAS of BRI in human subjects. Variants in asthma GWAS were significantly enriched for ASM QTLs and BRI-associated SNPs, and near genes enriched for ASM function, many with small *P* values that did not reach stringent thresholds of significance in GWAS.

**Conclusions:**

Our study identified significant differences between ASMCs from asthma cases and controls, potentially reflecting trained tolerance in these cells, as well as a set of variants, overlooked in previous GWAS, which reflect the AHR component of asthma.

## Background

Asthma is a common disease with a complex etiology, characterized by significant clinical and genetic heterogeneity. Moreover, estimates of heritability are approximately 0.50 [1], suggesting nearly equal genetic and environmental contributions to risk. In particular, reversible airway hyperresponsiveness (AHR), i.e., exaggerated bronchoconstriction (smooth muscle-mediated airway luminal narrowing), is a key feature of asthma. Although the precise mechanisms through which these functions occur are unknown, they are likely multifactorial, including genetic factors, inflammation, airway remodeling, and constitutive contractile dysfunction of airway smooth muscle cells (ASMCs). Cytokine-induced modification of ASMCs in the development of AHR has been extensively described (reviewed in [2–4]), even though the mechanisms through which these changes occur are not completely known. In particular, IL-13 [5–7] and IL-17 [8] act directly on ASM to potentiate contractile responses. Importantly, both of these cytokines have been implicated in asthma [4, 9], each defining important asthma endotypes: IL-13 is associated with the type 2 (T2)-high endotype, IL-17 with the Th17-high endotype, and IL-13+IL-17 with the mixed T2/Th17 endotype [10–12]. These combined data suggest that the micro-environment created by local immune responses in the lung of individuals with asthma potentiates AHR, and such responses may differ among individuals with the T2-high, the IL-17-high, and the mixed T2/Th17 asthma endotypes.

Genome-wide association studies (GWASs) have revealed many loci contributing to asthma risk, but post-GWAS challenges remain. For example, most of the variants identified in GWASs of asthma are located in non-coding regions of the genome and have unknown functions. Using bioinformatic predictions, recent large asthma GWASs have reported enrichments for SNPs located in regions with enhancer activity in immune cells [13–15] or with genes that are most highly expressed in skin, lung tissue, spleen, small intestine, and immune cells [16]. However, these inferences are limited to available resources, such as the Gene-Tissue Expression (GTEx) Consortium [17], the Roadmap Epigenomics [18], and the Encyclopedia of DNA Elements (ENCODE) [19], none of which include ASMCs. In addition, many variants with small *P* values that do not reach stringent criteria for significance in GWAS may also contribute to risk (i.e., the “mid-hanging fruit” [20]) or have context-dependent effects (i.e., genotype by environment interactions). One strategy to assign function to associated GWAS variants and to identify novel variants from among the mid-hanging fruit is to use cell cultures to model environment-specific responses in disease-relevant tissues and examine molecular and cellular endpoints.

Our results revealed that many asthma GWAS SNPs with small *P* values that do not reach thresholds of genome-wide significance reflect the smooth muscle component of asthma, highlighting the importance of using relevant tissues and exposures for fully characterizing the genetic architecture of asthma. We also observed profound response differences between cultured ASMCs from asthma cases and controls, suggesting that ASMCs have trained immunity [21], a sustained immune activation or tolerance (i.e., memory) to re-stimulation, likely due to epigenetic modifications that lead to long-lasting altered transcriptional responses, a feature previously described in leukocytes and coronary artery smooth muscle cells [22] that we now suggest for the first time in ASMCs. These combined results offer insights into the specific mechanisms that modulate, or lead to dysregulation of, smooth muscle phenotypes in asthma.

## Methods

### Experimental model and subject details

Primary airway smooth muscle cells were obtained through the Gift of Hope (GOH) Organ and Tissue Donor Network from human donor lungs that were not suitable for transplantation (Table 1). ASM cells from 75 donors were isolated from the trachea and main bronchi using established techniques [23]. Cells were split into a minimum of two tubes of one million cells each to be used for (1) contractility studies in Boston and (2) expression and methylation studies in Chicago. In both locations, frozen vials of cells were thawed and cultured in 75-cm^2^ flasks (see Additional File 1 for detailed methods). After 3 days, cells from each subject were transferred to NuSil-coated 96-well plates and cultured in serum-free media for 48 h, followed by 24-h exposure to IL-13 (10 ng/mL) (Peprotech), IL-17A (3 ng/mL) (Peprotech), both together, or vehicle control (10% FBS in PBS). Cytokine concentrations were selected for maximal contractile response (Additional File 1). After 24 h, lysates were collected and frozen at − 80 °C prior to RNA and DNA isolation in Chicago. In Boston, the cells were exposed to methacholine (Mch) after the 24-h treatment exposures, and then contractile responses were measured (see Additional File 1). Our study design is shown in Additional File 2.

**Table 1:** Characteristics of ASMC donors

	**With expression data**^**a**^	**With methylation data**^**a**^	**With contractile response data**	**With all three**
Sample size	67	70	65	63
Sex (males/females)	36/31	39/31	35/30	34/29
Ancestry (European American/African American)	50/17	51/19	48/17	47/16
Asthma^b^ (yes/no)	14/53	16/54	13/52	13/50
Median age at time of death (range) (years)	51 (21–85)	51 (21–85)	53 (21–85)	53 (21–85)
Ever smoker^c^ (%)	46 (69%)	49 (70%)	47 (72%)	46 (73%)
Median cell passage number (range)	4 (1–5)	4 (1–5)	4 (1–5)	4 (1–5)

^a^Includes only samples with genotype data

^b^Asthma was determined based on limited medical records and reports available at the time of death and was not the cause of death of any donor listed

^c^Smoking history reported after imputation, see Additional File 1 for details

#### DNA and RNA isolation

DNA for methylation studies and RNA for gene expression studies were isolated from cell lysates using the QIAgen AllPrep Kit (Qiagen). DNA for genotyping was isolated from untreated cells using the QIAamp DNA Blood Mini Kit (Qiagen).

### Quantification and statistical analysis

#### Genotyping and imputation of cell donors

DNA from 74 cell lines was genotyped using either the Illumina Omni2.5v8v1A or Human Core arrays. Genotypes within each platform were phased separately for European American and African American subjects using MACH [24] and imputed with minimac3 [25] using the 1000 Genomes phase 3 reference panels. Ancestry informative markers were used to determine ancestral PCs as described [26].

#### Gene expression and methylation analysis

RNA from vehicle and cytokine treated cells was hybridized to the Illumina Human HT-12 v4 array at The University of Chicago Functional Genomics Facility (FGF). Probes that were indistinguishable from background intensity (*P* < 0.01), contained more than one HapMap single nucleotide polymorphism (SNP), or mapped to multiple locations in the genome were removed. Since in some cases multiple probes mapped to one gene, median probe intensity was used to represent the transcriptional abundance of each individual transcript. Of the 47,231 transcripts on the Illumina Human HT12v4 array, 18,279 (39%) unique transcripts were detected as expressed in cultured ASMCs. DNA from vehicle and cytokine-treated cells was assessed for genome-wide methylation patterns using the Illumina Infinium Human MethylationEPIC Beadchip, also at the FGF.

Differential expression analyses between vehicle and cytokine-exposed cells as well as between individuals with and without asthma were performed in R (Version 1.0.136) using Limma [27, 28]. Because ancestry PC1 and PC2 captured the effects of global ancestry, they were included as covariates rather than self-reported race (i.e., African American vs. European American). Imputed smoking, age, and sex were included as covariates in all analyses. The final sample size for both analyses was 70 [29].

#### Sub-sampling analysis

To ensure that the differences in the gene expression and DNA methylation responses between individuals with and without asthma were not due to the differences in sample size, we randomly sub-sampled data from the 53 or 54, respectively, individuals without asthma to match the number of individuals with asthma for gene expression (*N* = 14) and DNA methylation studies (*N* = 16). We then analyzed 100 of these sub-sampled datasets using limma to detect differential expression or methylation following exposure to cytokines.

#### Molecular QTL mapping studies

Expression (e) QTL and methylation (me) QTL mappings were performed using matrix eQTL [30]. Windows of 500 kb from each transcription start site and 5 kb from each CpG were used for eQTL and meQTL mapping, respectively. To identify unique and shared QTLs across exposures, we selected QTLs at an FDR of 20% in each exposure as input into mashr [31], using a local false sign rate (lfsr) of 0.05. We classified 6390 eQTLs and 61,207 meQTLs as either shared or unique to the IL-13-, IL-17A-, and IL-13+IL-17A-exposed ASMCs at a local false sign rate (lfsr) of less than 5%. The vast majority of QTLs were shared across treatments, but three eQTLs (one gene) were unique to IL-17A-treated cells, and 780 meQTLs (291 CpGs) were unique to IL-13, 1672 meQTLs (597 CpGs) were unique to IL-17A, and 234 meQTLs (115 CpGs) were unique to IL-13+IL17A treatments, respectively.

#### Cellular mapping of contractile response (co) QTLs in ASMCs

A GWAS for contractile response was performed in ASMCs from 67 donors using GEMMA [32], including sex, age, ancestry PC1 and PC2, and smoking history as covariates. The resulting SNPs with *P* values < 0.01 were considered as coQTLs in subsequent analyses.

#### Physiologic (ph) QTL mapping of bronchial responsiveness index in the Hutterites

A GWAS for the quantitative trait bronchial responsiveness index (BRI) was performed in 964 Hutterite individuals using GEMMA [32]. Briefly, BRI was calculated from methacholine challenge studies (described in Motika et al. [33]) using the formula described in Burrows et al. [34]. QTL mapping was performed using a pedigree-based imputation program and variants from Hutterite whole-genome sequences [35, 36].

#### Enrichment analysis of QTLs in asthma GWASs

*P* values for each SNP were extracted from the largest GWAS to date for childhood-onset (< 12 years; *n* = 9433 cases and 318,237 controls) and adult-onset (26–75 years; *n* = 21,564 cases and 318,237 controls) asthma, which were conducted in the UK Biobank [16]. GARFIELD [37], an approach for functional enrichment analysis that corrects for linkage disequilibrium among SNPs, was used to assess enrichment of asthma GWAS SNPs with *P* values < 0.01 among all QTLs (eQTLs, meQTLs, coQTLs, and BRI GWAS SNPs).

#### Pathway analysis and enrichment testing

Protein-protein interaction network analyses were conducted using the Ingenuity Knowledge Base as implemented in Ingenuity Pathway Analysis (IPA; QIAGEN, https://www.qiagenbioinformatics.com/products/ingenuity-pathway-analysis/). Enrichment testing was performed using Advaita Bio’s iPathwayGuide (https://www.advaitabio.com/ipathwayguide). This software analysis tool implements the “impact analysis” approach that takes into consideration the direction and type of all signals on a pathway, the position, the role and type of every gene, etc., as described in [38–41]. A list of genes detected as expressed in ASMCs (*N* = 18,279) was used as the reference gene panel for all analyses.

## Results

**Fig. 1:**
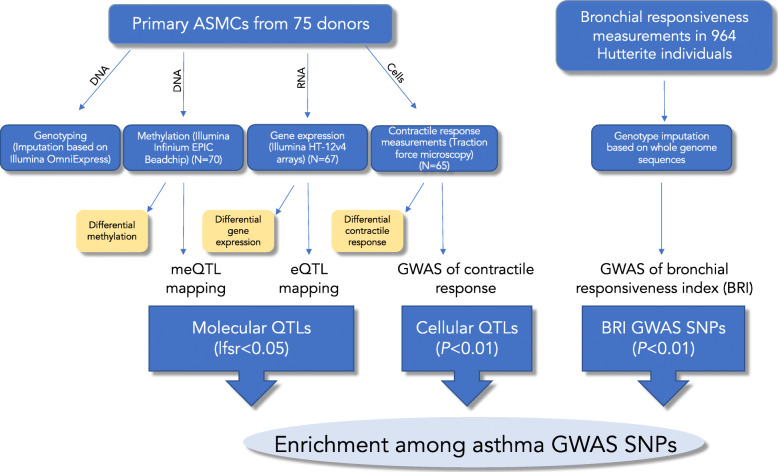
Study overview. Primary ASMCs from 75 donors were isolated in Chicago and then cultured in Chicago (genotyping, methylation, and expression studies) and Boston (contractile response measurements to methacholine) using identical protocols. In the final 24 h in both locations, cells were exposed to either vehicle, IL-13, IL-17A, or IL-13+IL17A. Genome-wide methylation and expression data were used to conduct differential response studies as well as to map molecular QTLs (eQTLs and meQTLs). Contractile response data were used as quantitative traits to investigate the differential response to cytokine exposure followed by methacholine exposure and to map cellular QTLs (coQTLs). The numbers of samples available for each assay after QC (with genotype data also available) are shown in parentheses. Independently, lung function studies were carried out in a human population. A quantitative measure of responsiveness to methacholine was assessed in these subjects (bronchial responsiveness index (BRI)), and a GWAS was performed. The resulting BRI GWAS SNPs, along with the molecular QTLs and cellular QTLs, were enriched among asthma GWAS SNPs

In this paper, we report the results of a novel cell model of AHR using primary airway smooth muscle cells exposed to IL-13 alone, IL-17A alone, and the combination of IL-13+IL-17A. As we describe below, we used this model to assess the (i) molecular (transcriptional and epigenetic) and cellular (contractility) responses of ASMCs to cytokine exposures, (ii) molecular response differences between cells from individuals with (cases) and without (controls) asthma, and (iii) molecular and cellular quantitative trait locus (QTL) mapping in these different environments. We also performed a GWAS of AHR in a human population. Finally, all four sets of associated variants were pooled and compared to a large GWAS of asthma. An overview of our study design is shown in Fig. 1.

### Differentially expressed genes in ASMCs exposed to asthma-promoting cytokines are enriched for genes near SNPs associated with asthma in GWAS

We used ASMCs collected from donor lungs, which were not used for transplant, in a cell culture model of response to cytokines. Cultured ASMCs exposed to IL-13 and/or IL-17A showed a robust transcriptional response to these asthma-promoting cytokines. Compared to vehicle exposure alone, IL-13 induced 4105 differentially expressed genes (DEGs), IL-17A exposure induced 1059 DEGs, and the combined treatment of IL-13+IL-17A induced 4519 DEGs at an FDR < 1%, among 18,279 genes detected as expression on the Illumina HT-12 v4 array (Additional File 3 and Additional File 4). A previous small study (*N* = 3 asthmatics and 3 controls) of IL-17A-exposed primary ASMCs [42] revealed similar DEGs in response to IL-17A as in our study (Additional File 5), suggesting that ASMC transcriptional responses to IL-17A are robust and reproducible, despite the differences in time point (2 h in [42] vs. 24 h in our study) and dosage (10 h in [42] vs. 3 ng/mL in our study).

**Fig. 2:**
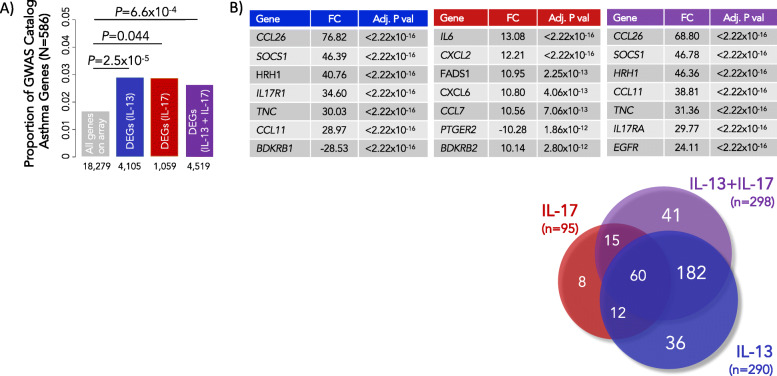
Asthma GWAS genes are over-represented among DEGs in response to IL-13 and/or IL-17A and among DEGs in cases compared to controls. In all panels, blue refers to IL-13 treated cells, red to IL-17A treated cells, and purple to IL-13+IL-17A treated cells. **a** DEGs following exposure to IL-13 and/or IL-17A were enriched for GWAS catalog genes near variants associated with asthma (*N* = 586) compared to all non-DEGs on the array. The numbers of DEGs for each exposure are shown on the *X*-axis, and the proportions of GWAS genes are shown on the *Y*-axis. **b** The 8 most significant DEGs are listed. The Venn diagram illustrates the total numbers and overlaps of DEGs that are also near SNPs highlighted in asthma GWASs

To assess the relevance of our cell culture model to asthma, we asked whether the DEGs in ASMCs were enriched for asthma genes named in the GWAS catalog [43]. These are genes near variants associated with asthma in previous GWAS, which we hereon refer to as asthma GWAS genes. To test for enrichment, we compared the proportion of asthma GWAS genes that were also DEGs for each exposure group to the proportion of asthma GWAS genes in all expressed transcripts. Indeed, DEGs in response to each treatment were enriched for asthma GWAS genes compared to all genes expressed on the array (Fig. 2a and Additional File 6). The eight most significant DEGs that are also asthma GWAS genes are shown in Fig. 2b. These results demonstrate that transcriptional responses to asthma-promoting cytokines in ASMCs alter genes near SNPs that have been replicated in many asthma GWASs, and provide tissue and context specificity to their roles in asthma.

**Fig. 3:**
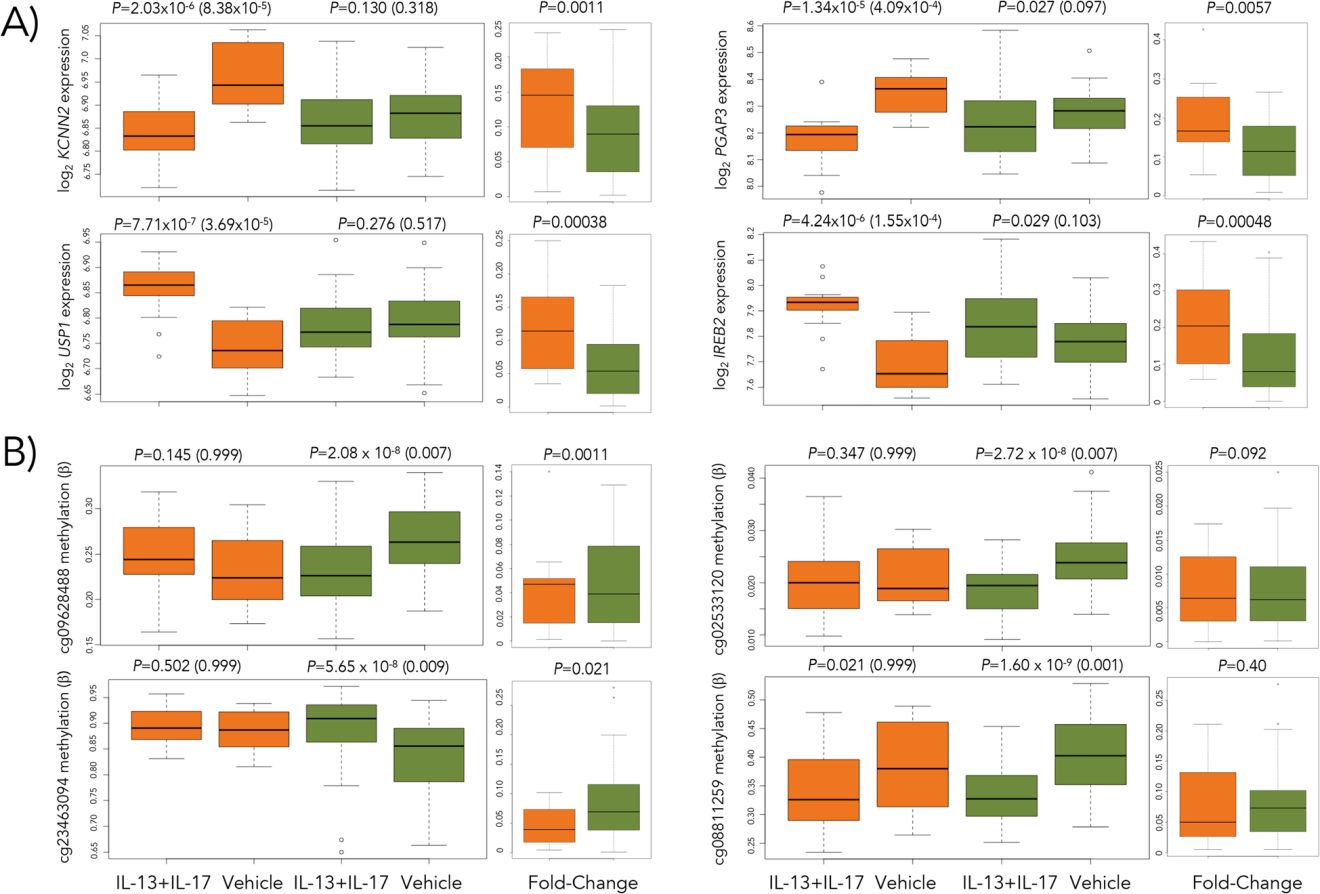
Transcription and methylation responses to cytokine exposure differ between ASMCs from cases and controls. **a** Four most significant differentially expressed genes (DEGs) in cases in response to IL-13+IL-17A compared to vehicle in ASMCs from individuals with (orange) and without (green) asthma. The fold change in transcription level from vehicle for cases and controls is shown to the right of each example. **b** Four most significant differentially methylated probes (DMPs) in response to IL-13+IL-17A compared to vehicle in ASMCs from individuals with (orange) and without (green) asthma. Different patterns are observed in response to cytokines between the two groups, including opposite effects, downregulation in response to IL-13+IL-17A in ASMCs from individuals without asthma only, upregulation in response to IL-13+IL-17A in ASMCs from individuals without asthma only, and similar trends but greater magnitudes of change among ASMCs from individuals without asthma. The fold change in the methylation level for each group is shown to the right of each example. *P* values are shown as unadjusted (adjusted) at the top of each panel. Sample sizes are *N* = 14 and 53 for cases and controls, respectively, in the expression studies and *N* = 16 and 54 for cases and controls in methylation studies

We next examined the transcriptional responses separately in ASMCs from asthma cases (*N* = 14) and controls (*N* = 53) (Additional File 7). Genes that were DE in one group (FDR < 5%) but not the other (FDR > 20%) are summarized and listed in Additional File 8. However, because the interpretation of differences in DEG numbers between cases and controls was confounded by differences in sample size, we randomly sampled 14 individuals from among the controls 100 times and performed an analysis of differential expression between the asthma cases and each of the 100 subsets of controls using a 2 × 2 interaction design in limma. Among the 100 sets of DEGs, we consistently observed more DEGs among the 14 cases than among the 100 random sets of 14 controls following exposure to IL-13, IL-17A, and IL-13+IL-17A compared to baseline (Mann-Whitney *U* test; *P* < 0.00001) (Additional File 7, panel D). Examples of the four most significant DEGs in ASMCs from asthma cases in response to IL-13+IL-17A are shown in Fig. 3a. These results indicate that transcriptional responses of airway smooth muscle cells are particularly sensitive to the effects of these asthma-promoting cytokines in individuals with asthma.

The 146 unique DEGs in ASMCs from asthma cases in response to IL-13 treatment were enriched for “ATF6-mediated unfolded protein response” (adj *P*_cases_ = 4.2 × 10^−5^, *P*_controls_ = 1.00) and “actin filament network formation” (adj *P*_cases_ = 0.046, *P*_controls_ = 0.25), while the 2585 unique DEGs in controls were enriched for “mitotic cell cycle processes” (adj *P*_controls_ = 4.2 × 10^−7^, *P*_cases_ = 1.00). The 298 unique DEGs from asthma cases in response to IL-13+IL-17A treatment were enriched for “cadherin binding” (adj *P*_cases_ = 9.0 × 10^−7^, *P*_controls_ = 0.067), while the 2717 unique DEGs from controls were enriched for “enzyme binding” (adj *P*_controls_ = 8.4 × 10^−4^, *P*_cases_ = 0.11). The DEGs in cases also formed two significant protein-protein interaction networks as revealed using IPA, one of which was enriched for “immunological disease” (score = 32) and one for “cell death and survival” (score = 32). Too few DEGs were observed among IL-17A-treated cells for enrichment or pathway analysis. Thus, the differential transcriptional responses to IL-13 and IL-13+IL-17A in ASMCs from asthma cases and controls were enriched for smooth muscle-specific processes and immune dysregulation, two cardinal features of asthma.

### DNA methylation responses to IL-13 and/or IL-17 are reduced in ASMCs from individuals with asthma

Unlike transcriptional responses to cytokines, we observed very few changes in methylation levels following exposure to IL-13 and/or IL-17A (Additional File 9). However, despite the lack of robust genome-wide methylation responses and in stark contrast to the gene expression studies, ASMCs from the asthma cases had fewer differentially methylated probes (DMPs) in response to the combined treatment of IL-13+IL-17A, with 0 DMPs observed in the cases and 260 DMPs observed in controls at an FDR < 5%. These differences remained at FDR thresholds as high as 20% (3 in cases and 6820 DMPs in controls). The mean number of DMPs among the 100 randomly selected subsets of 16 controls described above was 40.5 (SD = 106.1), still significantly more than the number of DMPs observed among the 16 cases (mean 2.9, SD = 2.3) (Mann-Whitney *P* < 0.00001) (Additional File 7, panel E; for examples, see Fig. 3b). The 225 genes nearest to the 260 DMPs in the controls were enriched for negative regulation of smooth muscle cell differentiation (adj *P* = 0.0001; GO:0051151). The enrichment for negative regulator genes in ASMCs from the controls suggests that ASM in individuals with asthma may have lost the ability to downregulate potentially pathogenic responses to immune modulators. Such epigenetic modifications in cells from asthma cases may have resulted from repeated exposures to AHR-promoting cytokines and suggest an epigenetic mechanism underlying the differing transcriptional responses of ASMCs between asthma cases and controls (see Additional File 10 for supporting data).

### Contractile responses correlate with transcriptional responses in IL-17A-exposed ASMCs

We assessed ASMC contractility as an in vitro cellular surrogate for AHR. Cells exposed to IL-17A showed the greatest response relative to vehicle, but we did not observe significant differences in smooth muscle contractile responses between cells from cases and controls (Additional File 11, panels A and B). To relate findings on DEGs following cytokine exposure to contractile response, we tested for correlations between transcript abundance and contractility in paired analyses of the 67 individuals in each of the treatment conditions (vehicle, IL-13, IL-17, and IL-13+IL-17). Although most of the correlation *P* values did not surpass correction for multiple testing (*P* = 2.73 × 10^−6^; corrected for 18,279 transcripts), we observed an excess of small correlation *P* values among IL-17A-exposed, but not among IL-13- or IL-13+IL-17A-exposed, ASMCs (*P* < 2.2 × 10^−6^; Kolmogorov-Smirnov test) (Additional File 11, panel C), indicating coordinated transcriptional and contractile responses of ASMCs to IL-17A in the absence of IL-13. The 433 genes most strongly correlated with ASMC contractile response in IL-17A-exposed cells (*P* ≤ 0.01) were enriched for “negative relaxation of cardiac muscle” (GO Biological Processes, adj *P* = 6.9 × 10^−4^), highlighting again the specificity of this response. These data suggest a more prominent role for IL-17A in potentiation of ASMC contractile response, which is tempered in the presence of IL-13 in the combined treatment model.

**Fig. 4:**
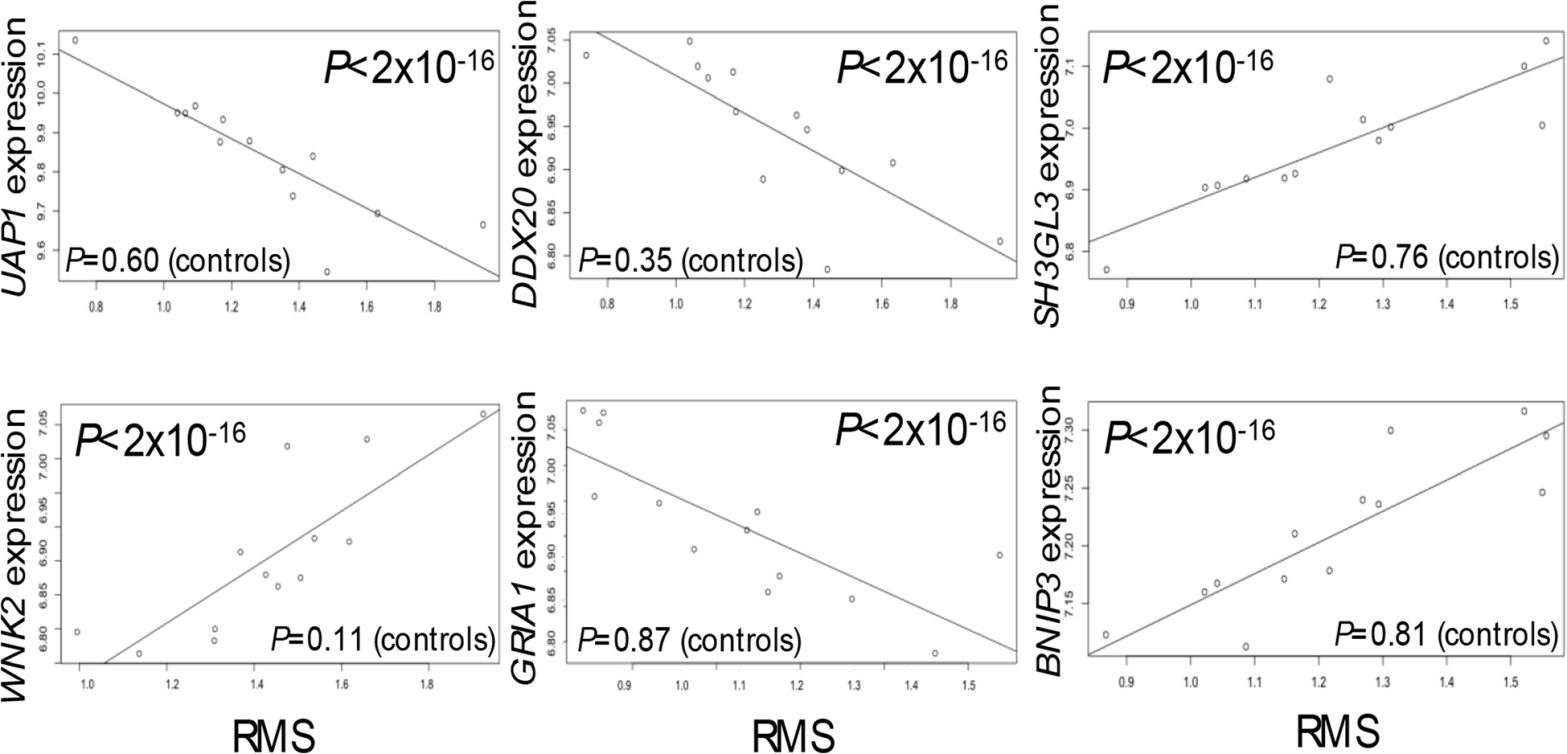
Example boxplots of significant correlations between RMS values (root mean square values measuring contractile response) and transcript levels. Spearman correlations were performed using RMS values and expression data from individuals with (*N* = 13) and without (*N* = 50) asthma separately. Only nine tests survived correction for multiple testing, and all were among ASMCs from donors with a history of asthma; six are shown here (*UAP1*, UDP-N-acetylglucosamine pyrophosphorylase 1; *DDX20*, DEAD-box helicase 20; *SH3GL3*, endophilin A3; *WNK2*, WNK lysine deficient protein kinase 2; *GRIA1*, glutamate ionotropic receptor AMPA type subunit 1; *BNIP3*, BCL2 interacting protein 3). *P* values in controls are shown along the bottom of each plot. Expression levels of the transcripts noted are shown on the *Y*-axis, and RMS values are plotted on the *X*-axis

Strikingly, when we perform this analysis separately in cells from asthma cases (*N* = 13) and controls (*N* = 50), all nine correlations between transcriptional and contractile responses that survived correction for multiple testing (FDR = 5%) were in the asthma cases (Fig. 4), despite the smaller number of cases and the observation that overall contractile responses were not significantly different between ASMCs from cases and controls. Two of the nine genes have been previously implicated in GWAS of lung function (endophilin A3; *SH3GL3* and glutamate ionotropic receptor AMPA type subunit 1; *GRIA1*) [44, 45], and a third (BCL2 interacting protein 3; *BNIP3*) has been implicated in bitter taste receptor agonist-induced ASM cell death [46]. These results further indicate that ASMCs from asthma cases and controls respond differently to IL-17A at both the transcriptional and cellular levels and suggest roles for novel gene targets in cytokine-enhanced smooth muscle contraction.

### A GWAS of BRI identifies candidate SNPs near genes relevant to ASM

In addition to our in vitro model of cellular and molecular responses to cytokines relevant to AHR, we performed a GWAS of bronchial responsiveness to Mch in a human population to provide complementary in vivo data. Briefly, response to methacholine was calculated in 964 Hutterite individuals, and a quantitative measure of response (bronchial responsiveness index (BRI)) was used in a GWAS (Additional File 12). Similar to the GWAS results of contractile response in ASMCs (see the “Methods” section), no signals reached genome-wide significance, and we therefore considered the 58,559 SNPs with *P* values < 0.01 as candidate BRI GWAS SNPs in subsequent analyses (Additional File 13).

### Molecular and cellular QTLs in cytokine-exposed ASMCs and SNPs from a GWAS of bronchial responsiveness in people are near genes enriched in immune and smooth muscle functions

To relate genetic variation that influences molecular and cellular responses to asthma-promoting cytokines to genetic variation associated with asthma in GWAS, we performed expression (e), methylation (me), and contractile (co) QTL mapping in cytokine-exposed ASMCs. The number of eQTLs and meQTLs identified at FDRs of 5% and the number of coQTLs with uncorrected *P* values < 0.01 in each treatment are shown in Table 2. The eQTL and meQTL results (FDR = 5%) are described in Additional Files 14 and 15, respectively; the coQTLs are described in Additional File 16. The overlap of the different categories of SNPs described above is shown in Additional File 17, and lists detailing the variants shared between the BRI GWAS and molecular QTLs and coQTLs are provided in Additional File 18.

**Table 2:** Results of eQTL and meQTL mapping using Matrix eQTL and coQTL discovery by GWAS. Numbers in parentheses for eQTLs and meQTLs refer to the number of unique genes (genes whose expression levels are associated with the QTL) and meCpGs (CpG sites whose methylation levels are associated with the QTL), respectively

**Category**	**Threshold**	**Vehicle**	**IL-13**	**IL-17A**	**IL-13+IL-17A**
eQTLs	FDR = 5%	2595 (140)	3262 (161)	2524 (133)	2765 (139)
meQTLs	FDR = 5%	10,797 (1718)	9859 (1515)	11,274 (1710)	13,222 (2046)
coQTLs	*P* < 0.01	9087	8705	9776	9370

Sex, age, imputed smoking, and the first two ancestry PCs were included as covariates

### Asthma GWAS SNPs with small *P* values are enriched among ASM QTLs

To assess the broad contribution to asthma risk of molecular and cellular QTLs in ASMCs and physiologic airway QTLs in people, we pooled variants from four separate analyses: eQTLs and meQTLs with lfsr < 0.05 and coQTLs and BRI GWAS SNPs with *P* values < 0.01, as described above, and tested for enrichment of these ASM-related QTLs among GWAS SNPs for childhood-onset and adult-onset asthma [16] using GARFIELD [37]. This revealed an enrichment of childhood-onset asthma GWAS SNPs, but not adult-onset GWAS SNPs, among ASM QTLs (Table 3). The contribution of the different QTLs to the 99 loci detected at a GWAS threshold of *P* < 0.01 is shown in Table 4; meQTLs (52%), coQTLs (16%), and BRI GWAS SNPs (30%) contributed to this enrichment, while only one eQTL was among them. It is important to note, however, that only the strongest QTL signal is considered by GARFIELD, and in many cases, there were multiple QTLs contributing to a locus. For example, an meQTL provided the strongest signal near ankyrin repeat and SOCS box containing 3 (*ASB3*), a gene that was recently reported to be associated with bronchodilator response during childhood and adolescence [47], but four other variants in linkage disequilibrium with the lead SNP also contributed to the signal, and all four of those SNPs are both meQTLs and coQTLs (see Additional File 19).

**Table 3:** Airway smooth muscle QTLs are enriched for asthma GWAS SNPs. Results of enrichment testing for molecular QTLs (lfsr < 0.05), cellular QTLs (*P* < 0.01), and BRI GWAS SNPs (*P* < 0.01) among GWAS SNPs with small *P* values (*P* < 0.01) using GARFIELD [37]

GWAS (Pividori et al. [16])	Enrichment *P* value^a^	GWAS *P* value threshold^b^	Fold enrichment	Number of lead SNPs
A) Adult-onset asthma (26–65 years) (21,564 cases and 318,237 controls)	0.38	0.01	0.87	59
	0.88	0.001	0.95	12
	0.38	1 × 10^−4^	0.41	1
B) Childhood-onset asthma (< 12 years) (9433 cases and 318,237 controls)	9.96 × 10^−5^	0.01	1.57	99
	0.0031	0.001	2.06	31
	0.0018	1 × 10^−4^	2.62	13

^a^Refers to the *P* value of the enrichment at the corresponding GWAS *P* value threshold (third column)

^b^The three significant enrichments (i.e., at GWAS *P* value thresholds of 0.01, 0.001, and 1 × 10^−4^) in the childhood-onset asthma GWAS are shown; the same three thresholds are shown for the adult-onset GWAS for comparison, despite the lack of significant enrichments

**Table 4:** Source of QTLs for the 99 lead loci identified by GARFIELD at an enrichment *P* value of 9.96 × 10^−5^

**QTL source**	**No. of lead loci**	**No. of loci by treatment**	**Treatment**^**a**^
meQTLs	52	13	Vehicle
		7	IL-13
		27	IL-17A
		5	IL-13+IL17A
eQTLs	1	1	IL-13+IL17A
coQTLs	16	2	Vehicle
		7	IL-13
		4	IL-17A
		3	IL-13+IL17A
BRI GWAS SNPs	30	30	NA
Total	99	99	

^a^In many cases, there were multiple QTLs contributing to a locus; only the exposure at which the smallest *P* value was observed is shown here (see Additional File 19 for more details)

Genes nearest ASMC QTLs that were also associated with asthma in the childhood-onset asthma GWAS were enriched for “SMAD protein phosphorylation” (adj *P* = 2.8 × 10^−4^) and “positive regulation of NK T cell differentiation” (adj *P* = 1.7 × 10^−4^), pathways with direct roles in smooth muscle contraction and AHR. Notably, all 99 lead SNPs for ASMC QTLs had *P* values that were below the threshold for genome-wide significance in the childhood-onset GWAS (range of *P* values from 9.7 × 10^−3^ to 3.43 × 10^−7^), falling into the category of mid-hanging fruit [20].

## Discussion

Airway smooth muscle contributes to asthma pathogenesis in multiple ways. The “twitchy” property that results in hypercontractile responses of smooth muscle cells in asthma defines airway hyperresponsiveness, which was the primary interest of our study, but structural changes and inflammatory signaling of ASMCs play important roles as well [48]. Despite the central role that smooth muscle plays in asthma, there has been relatively little focus on this cell type in genetic, genomic [49, 50], or epigenetic [51, 52] studies compared to other asthma-relevant cell types such as immune and airway epithelial cells [53–56]. The lack of comparable types of “omic” studies in ASMCs has made it impossible to attribute asthma GWAS SNPs to ASMC function or dysfunction. Here, we describe the first study to assess molecular and cellular QTLs in response to asthma- and AHR-promoting cytokines in ASMCs from the same individuals. These results, along with those of a GWAS of bronchial hyperresponsiveness, were integrated with results of large asthma GWASs to identify and functionally characterize variants contributing to childhood-onset and adult-onset asthma susceptibility. Including measurements of a contractile phenotype in cells from the same donors used for transcriptomic and epigenetic studies provided the opportunity to link genetic and molecular responses to a relevant cellular outcome and assess the effects of cytokine exposure directly on ASMC contraction.

Importantly, the relevance of our model to asthma is evidenced by three observations. First, transcriptional responses to asthma-promoting cytokines in ASMCs alter genes that are near highly replicated variants in asthma GWASs, potentially providing tissue and context specificity to their roles in asthma and validating this cell culture model for identifying genes, and potentially genetic variants, contributing to asthma susceptibility. Second, the excess of small *P* values for correlations between contractile and transcriptional responses in IL-17A-exposed cells highlights the specificity of this cytokine in ASMC contractility and therefore the importance of this model for studies of AHR. Finally, molecular and cellular QTLs identified using this in vitro model, together with SNPs identified in a GWAS of bronchial responsiveness, are enriched for asthma GWAS SNPs, confirming the importance of this model for annotating GWAS SNPs with regard to their potential effects on airway smooth muscle and asthma in people.

Linking SNPs associated with molecular responses to SNPs associated in GWAS can yield biological and clinical insights into genetic risks for disease. For example, three recent asthma GWASs reported enrichment of associated variants in specific target tissues. The TAGC GWAS [13] reported an enrichment of asthma-associated genetic variation in enhancer marks in immune cells based on data from the 111 Roadmap and 16 ENCODE reference epigenomes in 51 cells types [18]. A GWAS using UK Biobank data revealed that genes at childhood-onset loci were enriched for high expression in the skin, whereas adult-onset loci were enriched in the lung, using data on 53 tissues from up to 635 individuals included in GTEx [16], and a cross-trait analysis of asthma and allergic disease reported enrichments of GWAS loci in the skin, again using GTEx data [14]. Critically, these large, frequently cited databases do not include airway smooth muscle cells, nor do they include data on physiological response to asthma-relevant exposures. Placed in the context of these previous studies, the results of our study suggest that we are missing an important component of the genetic risk for asthma by ignoring the effect of genotype in disease-relevant tissues and physiological responses to disease-relevant exposures, such as contraction of airway smooth muscle cells in response to asthma-promoting cytokines. The practical implication of our results is that many SNPs associated with asthma in GWAS impact the expression of genes with functional (and possibly primary) roles in dysregulation of ASMCs in asthma pathogenesis.

The enrichment of asthma GWAS SNPs among ASM QTLs was observed only in the childhood-onset asthma GWAS, in which the genes at associated loci were enriched for overexpression in the skin [16], suggesting barrier function impairment as a primary etiology of asthma onset in childhood. We show that BRI GWAS SNPs are disproportionately overrepresented among the enriched childhood-onset GWAS SNPs (30 out of 99), highlighting again the lack of this important asthma cell type in large public databases. This relatively large overlap (~ 30%) between SNPs associated both with asthma and BRI GWAS SNPs is not necessarily surprising given that most individuals with asthma also have BHR, and the BRI is therefore strongly correlated with asthma, but the specificity of the signal for childhood-onset disease suggests that this reflects more than just a correlation with asthma, and may suggest cross-talk between the smooth muscle and epithelium in the lung in the context of barrier integrity. In support of this idea, many genes are involved in both barrier function and contractile processes, including myosin light chain kinase (*MLCK*) [57] and members of the Rho family of GTPases Rho and Rac [58, 59], further suggesting that the enrichment of asthma-associated SNPs among BRI-associated SNPs may highlight a set of genes with pleiotropic effects that influence asthma risk through both molecular pathways.

Our study also revealed significant transcriptional and epigenetic differences in cultured airway smooth muscle cells exposed to asthma-promoting cytokines between individuals with and without asthma. The increased transcriptional responses of ASMCs from individuals with asthma may be reflective of a higher sensitivity of these cells to asthma- and AHR-promoting cytokines due to chronic exposures in vivo, or may reflect genetic or other differences between ASMCs from asthma cases and controls.

Global changes in DNA methylation can reflect lifelong exposures to environmental variables, with responses potentially altering disease susceptibility through the modification of molecular phenotypes. In addition, epigenetic changes more generally have been implicated as the primary mechanism responsible for trained immunity in both immune and non-immune cells [21]. We had expected that exposure of ASMCs to IL-13 and/or IL-17A would result in appreciable changes to DNA methylation profiles, as seen with cultured bronchial epithelial cell responses to IL-13 exposure [55]. Instead, we observed very modest responses despite significant transcriptional changes in these same cells. It is possible, therefore, that ASMCs per se are not particularly sensitive to changes in DNA methylation or that a 24-h exposure of ASMCs to cytokines is not sufficient to induce methylation changes. Alternatively, the lack of epigenetic responses in ASMCs, particularly in those from individuals with asthma, may indicate that DNA methylation patterns in these cells are already fixed, due to either intrinsic defects or chronic exposure to IL-13 and IL-17A, to other mediators of the disease process, or to inhaled medication use. The fact that we observe enrichments for smooth muscle-specific processes among genes near meQTLs suggests that even modest changes in ASMC methylation, such as those due to nearby SNPs, may have an appreciable effect on smooth muscle phenotypes.

The diminished magnitude of an epigenetic (DNA methylation) response to cytokines in ASMCs from the cases, despite robust transcriptional responses in the cells from the same individuals and exposed to the same treatments, suggests that methylation profiles in these cells are established—and potentially dysregulated—in individuals with asthma. This observation is consistent with ASMCs from asthma cases having trained tolerance or memory-induced decreased responsiveness to inflammatory signals [21]. Although this phenomenon has not yet been documented in ASMCs, evidence for trained immunity in coronary artery SMCs has been reported [22]. The number of DEGs central to immune responses and the connection of these genes in a protein-protein interaction network enriched for “immunological disease” further suggests a prominent role for ASMCs in immune response processes.

The QTLs identified in this study reflect complementary aspects of airway smooth muscle function in asthma, none of which have previously been reported. The observation that ASM QTLs contribute specifically to risk for childhood-onset asthma suggests that specific mechanisms of susceptibility involve loci with a role in smooth muscle function that are involved in disease initiation as opposed to progression. The fact that the enriched ASM QTLs did not reach genome-wide significance in the GWAS further suggests that there may be significant heterogeneity with regard to primary ASM contributions among asthma cases and that some proportion of the missing heritability in asthma (as well as other complex diseases) is due to variants with exposure- or cell type-specific effects that may be difficult to detect in GWAS [20, 60]. Nonetheless, the observation that asthma GWAS SNPs among ASMC QTLs are enriched in SMAD protein phosphorylation-related pathways provides specificity to our results and further validates the critical, and previously unexplored, role of ASMCs in genetic risk for asthma.

Pathway enrichment results of genes with eQTLs suggest that whereas IL-13 may be more of an inducer of immune processes, consistent with the T2 endotype that has a significant allergic component, IL-17A may be more of an inducer of structural changes in ASMCs and subsequent predisposition to AHR, possibly resulting in more refractory asthma, consistent with the Th17 endotype. This observation from ASMCs is particularly interesting given that the T2 endotype has been defined and characterized in bronchial epithelial cells [61]. Overall, these combined studies point to the more prominent effects of IL-17A on transcriptional and epigenetic responses, the potentiation of contractile responses, and correlated changes in transcriptional and contractile responses in ASMCs.

There are a number of limitations to our study. First, while this study of genome-wide expression, methylation, and contractile responses in ASMCs is the largest to date, it is small for QTL studies, and especially for detecting differences between cells from individuals with and without asthma. Therefore, we likely missed many variants associated with gene expression, methylation, and contractile response or with differences between cases and controls. Second, we had limited clinical information on the lung donors, including age of asthma onset and medication use, and therefore could not directly assess associations between the molecular or cellular responses and clinical phenotypes beyond asthma in these individuals. Third, we used just one (24 h) time point to assess the response, and some relevant transcriptional, epigenetic, or contractile responses to cytokine exposures may occur at other time points. Finally, our cross-sectional study design does not allow us to ascribe the observed differences in transcriptional and epigenetic profiles between ASMCs from asthma cases and controls to those that were present prior to the onset of disease, and which could have therefore contributed to asthma risk by conferring a pre-existing enhanced response to asthma-promoting cytokines, from those that arose as a result of the disease process and permanently altered the response architecture in individuals with asthma. Nonetheless, despite these limitations, we have shown for the first time enrichments both for asthma-associated genes among IL-13 and/or IL-17A responsive genes in ASMCs and for GWAS variants associated with childhood-onset asthma among ASMC QTLs.

In summary, our study demonstrated that cytokine-exposed primary ASMCs can serve as a model for elucidating the function of asthma-associated GWAS SNPs and for prioritizing SNPs that do not reach stringent criteria for significance in GWAS for further studies. Considering the number of AHR-associated transcripts that are enriched in or specific to airway smooth muscle processes and QTLs enriched in asthma GWAS, we suggest that previous genetic studies of asthma missed an important subset of asthma risk genes. Our demonstration of genome-wide transcription, methylation, and contractile response differences between asthmatic and non-asthmatic ASMCs exposed to IL-13 and/or IL-17A highlights the importance of the context specificity of biological responses that underlie asthma disease processes and suggests that these cells show signatures of trained immunity. These observations combined with integrated analyses of ASM QTLs with asthma GWAS underscore the central role of airway smooth muscle in asthma pathogenesis and the utility of cell models of response in elucidating the function of GWAS SNPs.

## Conclusions

The lack of “omic” studies in ASMCs has made it impossible to attribute asthma GWAS SNPs to ASMC function or dysfunction. Here, we present the first study to assess molecular and cellular QTLs in response to asthma- and AHR-promoting cytokines in ASMCs from the same individuals. Our results demonstrate clear differences in patterns of genome-wide gene expression and methylation between cultured ASMCs from cases and controls, and offer insights into specific mechanisms that modulate, or lead to dysregulation of, smooth muscle phenotypes in asthma.

## Supplementary information

**Additional file 1.** Supplementary Methods. Additional details regarding cell culture conditions, data processing, and analysis.

**Additional file 2.** Overview of study design. An overview of the study design across two sites: Chicago and Boston.

**Additional file 3.** Volcano plots of transcriptional response to cytokines. Magnitude of transcriptional responses following 24 hours of exposure to cytokines.

**Additional file 4.** List of differentially expressed genes in response to cytokine exposure (FDR<1%). Transcripts differentially expressed in response to IL-13 and/or IL-17 compared to vehicle (FDR<1%).

**Additional file 5.** Comparison of transcriptional responses to IL-17 from another study. Genes reported by Dragon et al. to be up-regulated in response to IL-17 exposure.

**Additional file 6.** Differentially expressed genes that are also associated with asthma (GWAS catalog). Genes previously identified as being associated with asthma were compared to the list of genes differentially expressed in response to IL-13 and/or IL-17 compared to vehicle.

**Additional file 7. **A comparison of differentially expressed genes among individuals with and without asthma. Information regarding numbers of DEGs (FDR<5%) in response to IL-13, IL-17A, and IL-13+IL-17A among ASMCs from individuals with (*N*=14) and without (*N*=53) asthma, plus boxplots showing results of simulations to control for sample size between cases and controls.

**Additional file 8.** Genes that are DE in one group (cases or controls) that are not DE in the other group. Differentially expressed genes following cytokine exposure compared to baseline (vehicle) in cases or controls (FDR<5%) that are not DE (FDR>20%) in the opposite group.

**Additional file 9.** Volcano plots illustrating methylation responses to IL-13 and/or IL-17 in ASMCs. Methylation responses following 24 hours of exposure to IL-13, IL-17A, or IL-13+IL-17A compared to vehicle.

**Additional file 10.** Methylation levels among cases at baseline (vehicle) look more like controls following IL-13+IL-17 stimulation. Comparison of methylation levels at the 260 DMPs in controls following IL-13+IL-17 exposure to methylation levels among cases and controls at baseline (vehicle treated).

**Additional file 11.** Contractile measurements do not significantly differ between cases and controls or among treatment groups. Comparison of contractile measurements in IL-13 and/or IL-17A-exposed cells.

**Additional file 12. **Summaries of GWAS for bronchial responsiveness in the Hutterites. Q-Q plot of GWAS results for bronchical responsiveness index (BRI) in the Hutterites (*N*=964) and Manhattan plot of GWAS results for bronchical responsiveness index (BRI) in the Hutterites (N=964).

**Additional file 13. **Summary of BRI GWAS results. BRI GWAS SNPs (*P*<10^-5^) identified through GWAS of bronchial responsiveness index (BRI) in the Hutterites.

**Additional file 14.** List of eQTL results (FDR=5%). eQTLs identified in IL-13 and/or IL-17A-exposed ASMCs at an FDR of 5%.

**Additional file 15.** List of meQTL results (FDR=5%). meQTLs identified in IL-13 and/or IL-17A-exposed ASMCs at an FDR of 5%.

**Additional file 16. **List of coQTLs identified through GWAS in ASMCs. coQTLs (*P* < 0.001) identified through GWAS of contractile responses in cytokine- or vehicle-exposed ASMCs.

**Additional file 17. **Overlaps of QTLs and GWAS SNPs identified in this study. Venn diagram illustrating overlaps of molecular QTLs (e- and meQTLs; lfsr<0.05), BRI GWAS SNPs (*P*<0.01), and contractile response (co) QTLs (*P*<0.01).

**Additional file 18. **List of QTLs idenfitied in this study that are also BRI GWAS SNPs. eQTLs (lfsr<0.01), meQTLs (lfsr<0.05), and contractile response (co) QTLs (*P*<0.01) that are also BRI GWAS SNPs (*P*<0.01).

**Additional file 19.** Loci enriched in the UK Biobank childhood-onset asthma GWAS. Summary of the 99 loci enriched in UK Biobank childhood-onset asthma GWAS.

## Data Availability

The datasets generated and/or analyzed during the current study are available in public repositories: the Hutterite BRI GWAS summary data have deposited in dbGaP (phs000185.v7.p1) [36] (and is partially presented in Additional File 13), and the gene expression and methylation data have been deposited in GEO (Series GSE146377; https://www.ncbi.nlm.nih.gov/geo/query/acc.cgi?acc=GSE146377) [29]. The results for eQTL mapping (Additional File 14), meQTL mapping (Additional File 15), and coQTL mapping (Additional File 16) can all be found in the supplementary files.

## References

[1] Polderman TJ, Benyamin B, de Leeuw CA, Sullivan PF, van Bochoven A, Visscher PM (2015). Meta-analysis of the heritability of human traits based on fifty years of twin studies. Nat Genet.

[2] Amrani Y, Panettieri RA (2002). Modulation of calcium homeostasis as a mechanism for altering smooth muscle responsiveness in asthma. Curr Opin Allergy Clin Immunol.

[3] Shore SA (2004). Direct effects of Th2 cytokines on airway smooth muscle. Curr Opin Pharmacol.

[4] Yeganeh B, Xia C, Movassagh H, Koziol-White C, Chang Y, Al-Alwan L, et al. Emerging mediators of airway smooth muscle dysfunction in asthma. Pulm Pharmacol Ther. 2012;26(1):105–11. 10.1016/j.pupt.2012.06.011 22776693

[5] Risse PA, Jo T, Suarez F, Hirota N, Tolloczko B, Ferraro P, et al. Interleukin-13 inhibits proliferation and enhances contractility of human airway smooth muscle cells without change in contractile phenotype. Am J Physiol Lung Cell Mol Physiol. 2011. 10.1152/ajplung.00247.2010 21460123

[6] Tliba O, Deshpande D, Chen H, Van Besien C, Kannan M, Panettieri RA (2003). IL-13 enhances agonist-evoked calcium signals and contractile responses in airway smooth muscle. Br J Pharmacol.

[7] Chiba Y, Nakazawa S, Todoroki M, Shinozaki K, Sakai H, Misawa M (2009). Interleukin-13 augments bronchial smooth muscle contractility with an up-regulation of RhoA protein. Am J Respir Cell Mol Biol.

[8] Kudo M, Melton AC, Chen C, Engler MB, Huang KE, Ren X (2012). IL-17A produced by αβ T cells drives airway hyper-responsiveness in mice and enhances mouse and human airway smooth muscle contraction. Nat Med.

[9] Finkelman FD, Hogan SP, Hershey GK, Rothenberg ME, Wills-Karp M (2010). Importance of cytokines in murine allergic airway disease and human asthma. J Immunol.

[10] Bhakta NR, Erle DJ (2014). IL-17 and “TH2-high” asthma: adding fuel to the fire?. J Allergy Clin Immunol.

[11] Choy DF, Hart KM, Borthwick LA, Shikotra A, Nagarkar DR, Siddiqui S (2015). TH2 and TH17 inflammatory pathways are reciprocally regulated in asthma. Sci Transl Med..

[12] Robinson D, Humbert M, Buhl R, Cruz AA, Inoue H, Korom S (2017). Revisiting type 2-high and type 2-low airway inflammation in asthma: current knowledge and therapeutic implications. Clin Exp Allergy.

[13] Demenais F, Margaritte-Jeannin P, Barnes KC, Cookson WOC, Altmuller J, Ang W (2018). Multiancestry association study identifies new asthma risk loci that colocalize with immune-cell enhancer marks. Nat Genet.

[14] Zhu Z, Lee PH, Chaffin MD, Chung W, Loh PR, Lu Q (2018). A genome-wide cross-trait analysis from UK Biobank highlights the shared genetic architecture of asthma and allergic diseases. Nat Genet.

[15] Ferreira MA, Vonk JM, Baurecht H, Marenholz I, Tian C, Hoffman JD (2017). Shared genetic origin of asthma, hay fever and eczema elucidates allergic disease biology. Nat Genet.

[16] Pividori M, Schoettler N, Nicolae DL, Ober C, Im HK (2019). Shared and distinct genetic risk factors for childhood-onset and adult-onset asthma: genome-wide and transcriptome-wide studies. Lancet Respir Med.

[17] Consortium GT (2015). Human genomics. The Genotype-Tissue Expression (GTEx) pilot analysis: multitissue gene regulation in humans. Science..

[18] Roadmap Epigenomics C, Kundaje A, Meuleman W, Ernst J, Bilenky M, Yen A (2015). Integrative analysis of 111 reference human epigenomes. Nature..

[19] Consortium EP (2004). The ENCODE (ENCyclopedia Of DNA Elements) Project. Science..

[20] Ober C (2016). Asthma genetics in the post-GWAS era. Ann Am Thorac Soc.

[21] Hamada A, Torre C, Drancourt M, Ghigo E (2018). Trained immunity carried by non-immune cells. Front Microbiol.

[22] Schnack L, Sohrabi Y, Lagache SMM, Kahles F, Bruemmer D, Waltenberger J (2019). Mechanisms of trained innate immunity in oxLDL primed human coronary smooth muscle cells. Front Immunol.

[23] Panettieri RA (2001). Isolation and culture of human airway smooth muscle cells. Methods Mol Med.

[24] Li Y, Willer CJ, Ding J, Scheet P, Abecasis GR (2010). MaCH: using sequence and genotype data to estimate haplotypes and unobserved genotypes. Genet Epidemiol.

[25] Howie B, Fuchsberger C, Stephens M, Marchini J, Abecasis GR (2012). Fast and accurate genotype imputation in genome-wide association studies through pre-phasing. Nat Genet.

[26] Tandon A, Patterson N, Reich D (2011). Ancestry informative marker panels for African Americans based on subsets of commercially available SNP arrays. Genet Epidemiol.

[27] Phipson B, Lee S, Majewski IJ, Alexander WS, Smyth GK (2016). Robust hyperparameter estimation protects against hypervariable genes and improves power to detect differential expression. Ann Appl Stat.

[28] Ritchie ME, Phipson B, Wu D, Hu Y, Law CW, Shi W (2015). limma powers differential expression analyses for RNA-sequencing and microarray studies. Nucleic Acids Res..

[29] Thompson EE, Dang Q, Mitchell-Handley B, Rajendran K, Solway J, Ober C, et al. Cytokine-induced molecular responses in airway smooth muscle cells inform genome-wide association studies of asthma. GEO. 2020; https://www.ncbi.nlm.nih.gov/geo/query/acc.cgi?acc=GSE146377. 10.1186/s13073-020-00759-w PMC7370514 32690065

[30] Shabalin AA (2012). Matrix eQTL: ultra fast eQTL analysis via large matrix operations. Bioinformatics..

[31] Urbut SM, Wang G, Stephens M (2017). Flexible statistical methods for estimating and testing effects in genomic studies with multiple conditions.

[32] Zhou X, Stephens M (2012). Genome-wide efficient mixed-model analysis for association studies. Nat Genet.

[33] Motika CA, Papachristou C, Abney M, Lester LA, Ober C (2011). Rising prevalence of asthma is sex-specific in a US farming population. J Allergy Clin Immunol.

[34] Burrows B, Sears MR, Flannery EM, Herbison GP, Holdaway MD (1992). Relationships of bronchial responsiveness assessed by methacholine to serum IgE, lung function, symptoms, and diagnoses in 11-year-old New Zealand children. J Allergy Clin Immunol.

[35] Livne OE, Han L, Alkorta-Aranburu G, Wentworth-Sheilds W, Abney M, Ober C (2015). PRIMAL: fast and accurate pedigree-based imputation from sequence data in a founder population. PLoS Comput Biol.

[36] Thompson EE, Dang Q, Mitchell-Handley B, Rajendran K, Solway J, Ober C, et al. Cytokine-induced molecular responses in airway smooth muscle cells inform genome-wide association studies of asthma. dbGaP. 2020; https://www.ncbi.nlm.nih.gov/projects/gap/cgi-bin/study.cgi?study_id=phs000185.v7.p1. 10.1186/s13073-020-00759-w PMC7370514 32690065

[37] Iotchkova V, Ritchie GRS, Geihs M, Morganella S, Min JL, Walter K (2019). GARFIELD classifies disease-relevant genomic features through integration of functional annotations with association signals. Nat Genet.

[38] Ahsan S, Draghici S. Identifying significantly impacted pathways and putative mechanisms with iPathwayGuide. Curr Protoc Bioinformatics. 2017;57:7 15 1–7 30. 10.1002/cpbi.24 28654712

[39] Donato M, Xu Z, Tomoiaga A, Granneman JG, Mackenzie RG, Bao R (2013). Analysis and correction of crosstalk effects in pathway analysis. Genome Res.

[40] Draghici S, Khatri P, Tarca AL, Amin K, Done A, Voichita C (2007). A systems biology approach for pathway level analysis. Genome Res.

[41] Tarca AL, Draghici S, Khatri P, Hassan SS, Mittal P, Kim JS (2009). A novel signaling pathway impact analysis. Bioinformatics..

[42] Dragon S, Hirst SJ, Lee TH, Gounni AS (2014). IL-17A mediates a selective gene expression profile in asthmatic human airway smooth muscle cells. Am J Respir Cell Mol Biol.

[43] Buniello A, MacArthur JAL, Cerezo M, Harris LW, Hayhurst J, Malangone C, McMahon A, Morales J, Mountjoy E, Sollis E, Suveges D, Vrousgou O, Whetzel PL, Amode R, Guillen JA, Riat HS, Trevanion SJ, Hall P, Junkins H, Flicek P, Burdett T, Hindorff LA, Cunningham F and Parkinson H. The NHGRI-EBI GWAS Catalog of published genome-wide association studies, targeted arrays and summary statistics 2019. Nucleic Acids Research, 2019;47(D1005-D1012). 10.1093/nar/gky1120 PMC6323933 30445434

[44] Lutz SM, Cho MH, Young K, Hersh CP, Castaldi PJ, McDonald ML (2015). A genome-wide association study identifies risk loci for spirometric measures among smokers of European and African ancestry. BMC Genet.

[45] Wain LV, Shrine N, Artigas MS, Erzurumluoglu AM, Noyvert B, Bossini-Castillo L (2017). Genome-wide association analyses for lung function and chronic obstructive pulmonary disease identify new loci and potential druggable targets. Nat Genet.

[46] Pan S, Sharma P, Shah SD, Deshpande DA (2017). Bitter taste receptor agonists alter mitochondrial function and induce autophagy in airway smooth muscle cells. Am J Physiol Lung Cell Mol Physiol..

[47] Israel E, Lasky-Su J, Markezich A, Damask A, Szefler SJ, Schuemann B (2015). Genome-wide association study of short-acting beta2-agonists. A novel genome-wide significant locus on chromosome 2 near ASB3. Am J Respir Crit Care Med.

[48] Doeing DC, Solway J (2013). Airway smooth muscle in the pathophysiology and treatment of asthma. J Appl Physiol (1985).

[49] Himes BE, Qiu W, Klanderman B, Ziniti J, Senter-Sylvia J, Szefler SJ (2013). ITGB5 and AGFG1 variants are associated with severity of airway responsiveness. BMC Med Genet.

[50] Nieuwenhuis MA, Vonk JM, Himes BE, Sarnowski C, Minelli C, Jarvis D (2017). PTTG1IP and MAML3, novel genomewide association study genes for severity of hyperresponsiveness in adult asthma. Allergy..

[51] Yu Q, Yu X, Zhao W, Zhu M, Wang Z, Zhang J, et al. Inhibition of H3K27me3 demethylases attenuates asthma by reversing the shift in airway smooth muscle phenotype. Clin Exp Allergy. 2018;48(11):1439–52. 10.1111/cea.13244 30084510

[52] Perry MM, Lavender P, Kuo CS, Galea F, Michaeloudes C, Flanagan JM, et al. DNA methylation modules in airway smooth muscle are associated with asthma severity. Eur Respir J. 2018;51(4):1701068. 10.1183/13993003.01068-2017 PMC5902304 29449426

[53] Li X, Hastie AT, Hawkins GA, Moore WC, Ampleford EJ, Milosevic J (2015). eQTL of bronchial epithelial cells and bronchial alveolar lavage deciphers GWAS-identified asthma genes. Allergy..

[54] Luo W, Obeidat M, Di Narzo AF, Chen R, Sin DD, Pare PD (2016). Airway epithelial expression quantitative trait loci reveal genes underlying asthma and other airway diseases. Am J Respir Cell Mol Biol.

[55] Nicodemus-Johnson J, Naughton KA, Sudi J, Hogarth K, Naurekas ET, Nicolae DL (2016). Genome-wide methylation study identifies an IL-13-induced epigenetic signature in asthmatic airways. Am J Respir Crit Care Med.

[56] Xu CJ, Soderhall C, Bustamante M, Baiz N, Gruzieva O, Gehring U (2018). DNA methylation in childhood asthma: an epigenome-wide meta-analysis. Lancet Respir Med.

[57] Shen Q, Rigor RR, Pivetti CD, Wu MH, Yuan SY (2010). Myosin light chain kinase in microvascular endothelial barrier function. Cardiovasc Res.

[58] Vouret-Craviari V, Boquet P, Pouyssegur J, Van Obberghen-Schilling E (1998). Regulation of the actin cytoskeleton by thrombin in human endothelial cells: role of Rho proteins in endothelial barrier function. Mol Biol Cell.

[59] Wojciak-Stothard B, Potempa S, Eichholtz T, Ridley AJ (2001). Rho and Rac but not Cdc42 regulate endothelial cell permeability. J Cell Sci.

[60] Watanabe K, Umicevic Mirkov M, de Leeuw CA, van den Heuvel MP, Posthuma D (2019). Genetic mapping of cell type specificity for complex traits. Nat Commun.

[61] Woodruff PG, Modrek B, Choy DF, Jia G, Abbas AR, Ellwanger A (2009). T-helper type 2-driven inflammation defines major subphenotypes of asthma. Am J Respir Crit Care Med.

