# Supplementary material for: Cytokine-induced molecular responses in airway smooth muscle cells inform genome-wide association studies of asthma
Source: Genome Med. 2020 Jul 20;12:64. doi: 10.1186/s13073-020-00759-w (PMC7370514; doi:10.1186/s13073-020-00759-w)
Supplement: Supplementary file 1 — Additional file 1. Supplementary Methods. Additional details regarding cell culture conditions, data processing, and analysis. [file 13073_2020_759_MOESM1_ESM.pdf]

SUPPLEMENTARY METHODS

Experimental model and subject details

Primary airway smooth muscle cells were obtained through the Gift of Hope (GOH) Organ and Tissue Donor Network from human donor lungs that were not suitable for transplantation. ASM cells from 75 donors were isolated from trachea and main bronchi using established techniques (Panettieri, 2001). Cells were grown to 80% confluency, counted, and split into a minimum of two tubes of one million cells each to be used for 1) contractility studies in Boston and 2) expression and methylation studies in Chicago. In both locations, frozen vials of cells were thawed and cultured in 75 cm^2 flasks in DMEM/F-12 media (Invitrogen) supplemented with 10% FBS, 5% non-essential amino acids (Invitrogen), and 5% antibiotic/antimycotic (Invitrogen). After 3 days, cells from each subject were trypsinized, counted, and transferred to NuSil-coated 96 well plates, with the locations of each treatment changing from one batch to the next to avoid edge or plate effects. Cells were cultured in quadruplicate wells in serum free media (F12 supplemented with 1% pen/strep (Sigma), 1% glutamine (Invitrogen), 1% fungizone (Invitrogen), 1.2% 1M NaOH, 0.17% $CaCl_2*2H_2O$, and 1% insulin-transferrin-selenium (ITS; Life Technology/Gibco) for 48 hours, followed by 24-hour exposure to IL-13 (10 ng/mL) (Peprotech), IL-17A (3 ng/mL) (Peprotech), both together, or vehicle control (10% FBS in PBS). Cytokine concentrations were selected for maximal contractile response based on pilot studies. After 24 hours, wells were washed with PBS and lysis buffer was added. Lysates were collected and cells from each individual were pooled by treatment, and frozen at -80°C prior to RNA and DNA isolation in Chicago. In Boston, the cells were exposed to methacholine (Mch) after the 24-hour treatment exposures and then contractile responses were measured. Cells were cultured at

37°C at all times and authenticated by visual inspection. Cells from one donor were lost to contamination in Chicago and cells from four donors were lost to contamination in Boston. Because materials used in this study were obtained from deceased subjects, The Institutional Review Board at the University of Chicago does not consider them human subjects.

Method Details

Contractility Studies in Cultured ASMCs

Rectangular glass slides (55mm x 75mm x 0.1mm) were cleaned with acetone, then with isopropanol. A first layer of a silicone-based polymer, NuSil (NuSil Silicone Technologies) with an elastic modulus of 12.1kPa and a thickness of approximately 100 μm was uniformly spread on top of the beads, covering the slide. The NuSil layer was incubated at 100°C for 1 hour in order to cure. A second layer of NuSil also with an elastic modulus of 12.1kPa but with a thickness of approximately 1 μm was then spin coated onto the first layer. Pre-mixed into this second layer was a 1% volume fraction of ~400 nm size green-fluorescence microspheres. In this manner, the final composite (layer 1 + layer 2) yielded a substrate with a surface layer of finely dispersed fluorescent microbeads. The composite was then incubated at 100°C for 1 hour in order to cure. Separately, 55mm x 75mm x 10mm molds of patterned 96-well plastic were coated with the silicone elastomer, Sylgard (Dow Corning) and lightly pressed against NuSil to strongly bond to it. Each hole from the patterned slab functioned as an individual well, with the NuSil substrate within each well.

Fourier transform traction microscopy (FTTM) was performed in each well of the multi-well plate. An image of surface microspheres (green beads) and of cells were obtained in quick succession. Such image pairs were registered at three different time points: before cell plating (reference), at the pre-methacholine baseline (baseline), and during methacholine exposure (during

Mch). By comparing the basal or treated position of surface beads with the corresponding reference position, the cell-exerted displacement field at baseline and during Mch exposure were obtained (Trepat et al., 2009). From the displacement field and from knowledge of substrate stiffness and substrate thickness, the monolayer traction field and the root mean squared (RMS) value of traction were computed (Butler et al., 2002, Trepat et al., 2009).

DNA and RNA Isolation

DNA for methylation studies and RNA for gene expression studies were isolated from cell lysates using the QIAgen AllPrep Kit (Qiagen). DNA for genotyping was isolated from untreated cells using the QIAamp DNA Blood Mini Kit (Qiagen).

Quantification and Statistical Analysis

Genotyping and imputation of cell donors

DNA from 74 cell lines was genotyped using either the Illumina Omni2.5v8v1A or Human Core arrays. All individuals had call rates >98%. After extracting the set of overlapping variants, a total of 236,843 with call rates >98% remained. Two pairs of subjects were determined to be identical to one another, and two failed sex checks. The samples that failed sex checks and one of each duplicated pair were dropped from all downstream analyses yielding a final sample size of 70 unrelated subjects.

Genotypes within each platform were phased separately for European American and African American subjects using MACH (Li et al., 2010) and imputed with minimac3(Howie et al., 2012) using the 1,000 Genomes phase 3 reference panels. SNPs with an imputation efficiency >0.7 within each ethnicity/platform analysis were retained, and biallelic variants with

a MAF > 10% in both the European American and African American samples were used in subsequent studies (n=1,005,490). Ancestry informative markers were used to determine ancestral PCs as described (Tandon et al., 2011).

Gene Expression Analysis

RNA from vehicle and cytokine treated cells was hybridized to the Illumina Human HT-12 v4 array at The University of Chicago Functional Genomics Facility. All samples had RIN scores ≥9.7 with the exception of one (<3), which was not sent for processing; the three other conditions for that individual were also excluded. The probe level raw intensity values across arrays were normalized using quantile normalization and background corrected normalized expression values were obtained for each probe using the R package lumi (Du et al., 2008). Probes that were indistinguishable from background intensity ($P<0.01$), contained more than one HapMap single nucleotide polymorphism (SNP), or mapped to multiple locations in the genome (Nicodemus-Johnson et al., 2016) were removed. Median probe intensity was used to represent the transcriptional abundance of each gene. Of the 47,231 transcripts on the Illumina Human HT12v4 array, 18,279 (39%) were detected as expressed in cultured ASMCs.

The strongest effects on expression variability were treatment; extraction batch, chip, RNA concentration, cell line age (i.e. number of months frozen), smoking history, and plate as identified by principal components analysis (PCA) (Leek et al., 2010) analysis of the gene expression data. The technical effects of culture and extraction batch, chip and plate were removed using ComBat (Johnson et al., 2007), and RNA concentration and cell line age were removed using linear regression. Ancestry PC1 and PC2 were included as covariates. History of smoking, age and sex were included as covariates in all analyses. The final sample size for gene

expression analyses was 70. Differential expression analyses between individuals with and without asthma were performed in R (Version 1.0.136) using Limma (Phipson et al., 2016, Ritchie et al., 2015) using a random effects model where individual IDs were coded as random effects.

Methylation Analysis

DNA from vehicle and cytokine treated cells was assessed for genome-wide methylation patterns using the Illumina Infinium Human MethylationEPIC Beadchip at The University of Chicago Functional Genomics Facility. Probes located on the sex chromosomes and those with detection P values > 0.01 in 75% of samples were removed. Probes mapping to more than one genomic location or overlapping with known SNPs (MAF>5% in either African Americans or European Americans) were also excluded (McCartney et al., 2016). Data were processed using Minfi (Aryee et al., 2014); Infinium type I and type II probe bias were corrected using SWAN (Maksimovic et al., 2012). Raw probe values were corrected for color imbalance and background by control normalization.

Data quality was assessed using PCA. One sample was an outlier in PCA analysis (PC3 and PC4) and was removed. The final sample size for methylation studies was 70. Culture and extraction batch, array, and cell passage number were removed using ComBat, and DNA concentration and cell line age were removed using linear regression. Sex and age were significant variables and were included as covariates in all analyses; imputed smoking was also included as a covariate. DNA methylation levels are reported as β values at each CpG site, which is the fraction of signal obtained from the methylated beads relative to the sum of methylated and

unmethylated bead signals. Differential methylation analyses between individuals with and without asthma were performed in R using Limma.

Subsampling analysis

To ensure that the differences in gene expression and DNA methylation responses between individuals with and without asthma were not due to differences in sample size, we randomly sub-sampled data from the 53 or 54, respectively, individuals without asthma to match the number of individuals with asthma for gene expression (N=14) and DNA methylation studies (N=16). We then analyzed 100 of these sub-sampled datasets using limma to detect differential expression or methylation following exposure to cytokines.

Imputing smoking status

The clinical variable with the greatest amount of missing data (N=16) was smoking history (defined as 'smoking ever'). Of the remaining sample, 35 donors were classified as ever-smokers and 19 as never-smokers. To assess whether genome-wide methylation levels in ASMCs could be used to impute smoking status, we removed individuals with missing data and performed differential methylation analysis between smokers and non-smokers. We identified 440 CpGs (FDR=5%) that were differentially methylated between individuals with and without a history of smoking. We then performed hierarchical clustering analysis of the samples based on those DMCs. This yielded an epigenetic prediction of smoking history with 89% sensitivity and 95% specificity. We then included the 440 predictive CpG sites from the 16 subjects missing smoking status in the hierarchical clustering analysis and assign imputed smoking history to

these subjects. Imputed smoking status was included as covariates in all downstream analyses of gene expression, DNA methylation, and contractility.

Molecular QTL mapping studies

Expression (e)QTL and methylation (me)QTL mapping were performed using matrix eQTL(Shabalin, 2012). Windows of 500 kilobase (kb) from each transcription start site and 5 kb from each CpG were used for eQTL and meQTL mapping, respectively. For both studies, age, sex, imputed smoking history, and the first two ancestry PCs were used as covariates. To identify unique and shared QTLs across exposures, we selected QTLs at an FDR of 20% in each exposure as input into mashr (Urbut et al., 2019), using a local false sign rate (lfsr) of 0.05. Using mashr, we classified 6,390 eQTLs and 61,207 meQTLs as either shared or unique to the IL-13, IL-17A, and IL-13+IL-17A-exposed ASMCs. The vast majority of QTLs were shared across treatments, but three eQTLs were unique to IL-17A treated cells, whereas 780, 1,672, and 234 meQTLs were unique to IL-13, IL-17A or IL-13+IL17A treatments, respectively.

Cellular (ce)QTL mapping of contractile response in ASMCs

A GWAS for contractile response was performed in ASMCs from the 67 donors using GEMMA (Zhou and Stephens, 2012), and including sex, age, ancestry PC1 and PC2, and smoking history as covariates.

QTL mapping of bronchial responsiveness index (BRI) in the Hutterites

A GWAS for the quantitative trait bronchial responsiveness index (BRI) was performed in 964 Hutterite individuals using GEMMA (Zhou and Stephens, 2012). Briefly, BRI was calculated from methacholine challenge studies (described in Motika et al. (Motika et al., 2011)) using the formula described in Burrows et al. (Burrows et al., 1992). QTL mapping was performed using a pedigree-based imputation program and variants from Hutterite whole genome sequences (Livne et al., 2015). Because variants were imputed based on pedigree information, missing genotype data reflect the absence of pedigree information and not quality of the genotypes. Therefore, we included SNPs with call rates ≥ 85% and MAF ≥ 0.05, similar to our previous studies in this population (Igartua et al., 2017, Mozaffari et al., 2018, Mozaffari et al., 2019). This yielded 5,358,732 variants for the BRI GWAS. Associations between genotype and inverse transformed phenotype were tested, including age and height as covariates; kinship coefficients between all pairs of individuals were included as a random effect to correct for relatedness between subjects.

Enrichment analysis of QTLs in asthma GWASs

P-values for each SNP were extracted from the largest GWAS to date for childhood-onset (<12 years; n=9,433 cases and 318,237 controls) and adult-onset (26-75 years; n=21,564 cases and 318,237 controls) asthma, which were conducted in the UK Biobank (Pividori et al., 2018). GARFIELD (Iotchkova et al., 2019), an approach for functional enrichment analysis that corrects for linkage disequilibrium among SNPs, was used to assess enrichment of asthma GWAS SNPs with *P*-values <0.01 among all QTLs (eQTLs, meQTLs, coQTLs) and BRI GWAS SNPs. meQTLs (52%), coQTLs (16%) and BRI-associated SNPs (30%) contributed to the observed enrichment among childhood-onset associated SNPs, while only one eQTL was among them. Only the strongest QTL signal is considered by GARFIELD and in many cases there were

multiple QTLs contributing to a locus. For example, an meQTL provided the strongest signal near ankyrin repeat and SOCS box containing 3 (*ASB3*), a gene that was recently reported to be associated with bronchodilator response during childhood and adolescence (Israel et al., 2015), but four other variants in linkage disequilibrium with the lead SNP also contributed to the signal, and all four of those SNPs are both meQTLs and coQTLs.

Pathway analysis and enrichment testing

Protein-protein interaction network analyses were conducted using the Ingenuity Knowledge Base as implemented in Ingenuity Pathway Analysis (IPA; QIAGEN, https://www.qiagenbioinformatics.com/products/ingenuity-pathway-analysis/). Network interactions were limited to those known to occur in primary cells or tissues; all other settings were left as the defaults. The score of each network is based on the network hypergeometric distribution and is calculated with the right-tailed Fisher's Exact Test to identify over-representation of genes in the input gene list relative to all genes present on the Illumina HT12 v4 array.

Enrichment testing was performed using Advaita Bio's iPathwayGuide (https://www.advaitabio.com/ipathwayguide). This software analysis tool implements the 'Impact Analysis' approach that takes into consideration the direction and type of all signals on a pathway, the position, role and type of every gene, etc., as described in (Ashan and Draghici, 2017; Donato et al., 2013; Draghici et al., 2007; Tarca et al., 2009). A list of genes detected as expressed in ASMCs (N=18,279) was used as the reference gene panel for all analyses.

Panettieri RA, Jr. Isolation and culture of human airway smooth muscle cells. Methods Mol Med. 2001; 56:155-60.

Trepat X, et al. Physical forces during collective cell migration. Nature Physics. 2009; 5:426-30.

Butler JP., et al. Traction fields, moments, and strain energy that cells exert on their surroundings. J Physiol Cell Physiol. 2002; 282:C595-605.

Li Y et al., MACH: Using sequence and genotype data to estimate haplotypes and unobserved genotypes. Genetic Epidemiology 2010; 34:816-34.

Tandon A et al., Ancestry informative marker panels for African Americans based on subsets of commercially available SNP arrays. Genet Epidemiol. 2011; 35:80-3.

Du P et al., lumi: a pipeline for processing Illumina microarray. Bioinformatics 2008; 24:1547-8.

Nicodemus-Johnson J et al., Genome-Wide Methylation Study Identifies an IL-13-induced Epigenetic Signature in Asthmatic Airways. Am J Respir Crit Care Med. 2016; 193:376-85.

Leek JT et al., Tackling the widespread and critical impact of batch effects in high-throughput data. Nature Reviews Genetics 2010; 11:733-39.

Johnson WE et al., Adjusting batch effects in microarray expression data using empirical Bayes methods. Biostatistics 2007; 8:118-27.

Phipson B et al., Robust hyperparameter estimation protects against hypervariable genes and improves power to detect differential expression. Ann Appl Stat. 2016; 10:946-63.

Ritchie ME et al., limma powers differential expression analyses for RNA-sequencing and microarray studies. Nucleic Acids Res. 2015; 43:e47.

McCartney DL et al., Identification of polymorphic and off-target probe binding sites on the Illumina Infinium MethylationEPIC BeadChip. Genom Data 2016; 9:22-4.

Aryee MJ et al., Minfi: a flexible and comprehensive Bioconductor package for the analysis of Infinium DNA methylation arrays. Bioinformatics 2014; 30:1363-9.

Maksimovic J et al., SWAN: Subset-quantile within array normalization for Illumina Infinium HumanMethylation450 BeadChips. Genome Biology; 13:R44.

Urbut SM et al., Flexible statistical methods for estimating and testing effects in genomic studies with multiple conditions. Nature Genetics 2019, 51:187-95.

Zhou X and Stephens M. Genome-wide efficient mixed-model analysis for association studies. Nat Genet. 2012; 44:821-4.

Motika CA et al., Rising prevalence of asthma is sex-specific in a US farming population. J Allergy Clin Immunol. 2011; 128:774-9.

Burrows B et al., Relationships of bronchial responsiveness assessed by methacholine to serum IgE, lung function, symptoms, and diagnoses in 11-year-old New Zealand children. J Allergy Clin Immunol. 1992; 90:376-85.

Livne OE et al. PRIMAL: Fast and accurate pedigree-based imputation from sequence data in a founder population. PLoS Comput Biol. 2015; e1004139.

Igartua C et al., Host genetics variation in mucosal immunity pathways influences the upper airway microbiome. Microbiome, 2017; 5:16.

Mozaffari SV et al., Parent of origin gene expression in a founder population identifies two new candidate imprinted genes at known imprinted regions. PLoS One, 2018; 13:e0203906.

Mozaffari SV et al., Parent of origin effects on quantitative phenotypes in a large Hutterite pedigree. Communications Biology, 2019; 2:28.

Pividori M et al., Shared and distint genetic risk factors for childhood onset and adult onset asthma. The Lancet, 2019; 7:P509-22.

Iotchkova V et al., GARFIELD classifies disease-relevant genomic features through integration of functional annotations with association signals. Nat Genet. 2019; 51:343-53.

Israel E et al., Genome-wide association study of short-acting b2-agonists: a novel genome-wide significant locus on chromosome 2 near ASB3. Am J Respir Crit Care Med 2015; 191:530-7.

Ahsan S and Draghici S. Identifying Significantly Impacted Pathways and Putative Mechanisms with iPathwayGuide. Curr Protoc Bioinformatics. 2017; 57:7 15 1-7 15 30.

Donato M et al., Analysis and correction of crosstalk effects in pathway analysis. Genome Research 2013; 23:1885-93.

Draghici S et al., A systems biology approach for pathway level analysis. Genome Research 2007; 17:1537-45.

Tarca AL et al., A novel signaling pathway impact analysis. Bioinformatics. 2009; 25:75-82.
