# Supplementary material for: Cytokine-induced molecular responses in airway smooth muscle cells inform genome-wide association studies of asthma
Source: Genome Med. 2020 Jul 20;12:64. doi: 10.1186/s13073-020-00759-w (PMC7370514; doi:10.1186/s13073-020-00759-w)

Additional File 2. Overview of study design. Cells were obtained from Gift of Hope donor individuals, and grown to ~80 confluency in Chicago. Cells were split into two vials, one of which was shipped to Boston and one remained in Chicago. Cells underwent identical culture protocols at both sites: ASMCs were plated in quadruplicate on 96-well plates upon nearing confluency in T75 flasks. Following 48 hours of serum deprivation, cells were exposed to IL-13, IL-17A, IL-13 + IL-17A, or vehicle control. After 24 hours, contractile responses were measured in Boston and cell lysates were collected and frozen at (-80°C) in Chicago for subsequent DNA and RNA isolation.

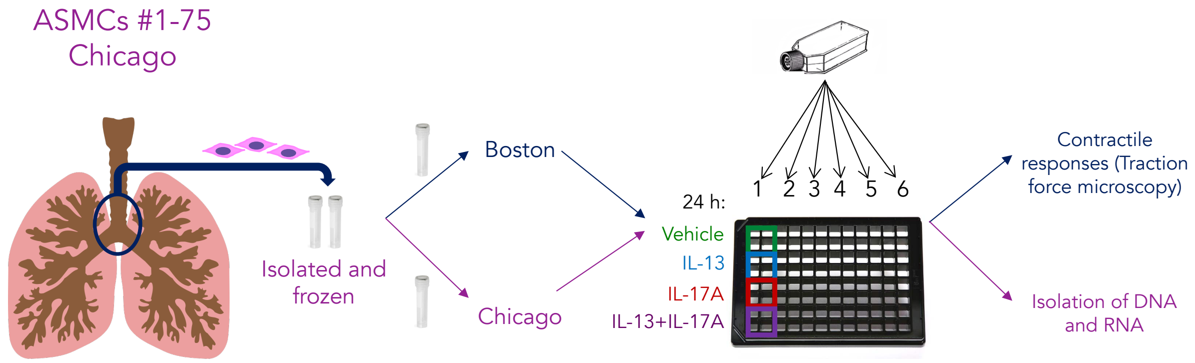

Supplement: Supplementary file 2 — Additional file 2. Overview of study design. An overview of the study design across two sites: Chicago and Boston. [file 13073_2020_759_MOESM2_ESM.pdf]
