# Supplementary material for: Cytokine-induced molecular responses in airway smooth muscle cells inform genome-wide association studies of asthma
Source: Genome Med. 2020 Jul 20;12:64. doi: 10.1186/s13073-020-00759-w (PMC7370514; doi:10.1186/s13073-020-00759-w)
Supplement: Supplementary file 3 — Additional file 3. Volcano plots of transcriptional response to cytokines. Magnitude of transcriptional responses following 24 hours of exposure to cytokines. [file 13073_2020_759_MOESM3_ESM.pdf]

Additional File 3. Magnitude of transcriptional responses following 24 hours of exposure to cytokines. Volcano plots showing magnitude of transcriptional response following exposure to IL-13, IL-17A, or IL-13+IL-17A compared to vehicle. Colored dots represent transcripts DE at FDR<1%; dots in black are not significant in each panel. Log_2(fold change) is plotted along the X-axis and $-log_{10}$(*P*-value) is plotted on the Y-axis.

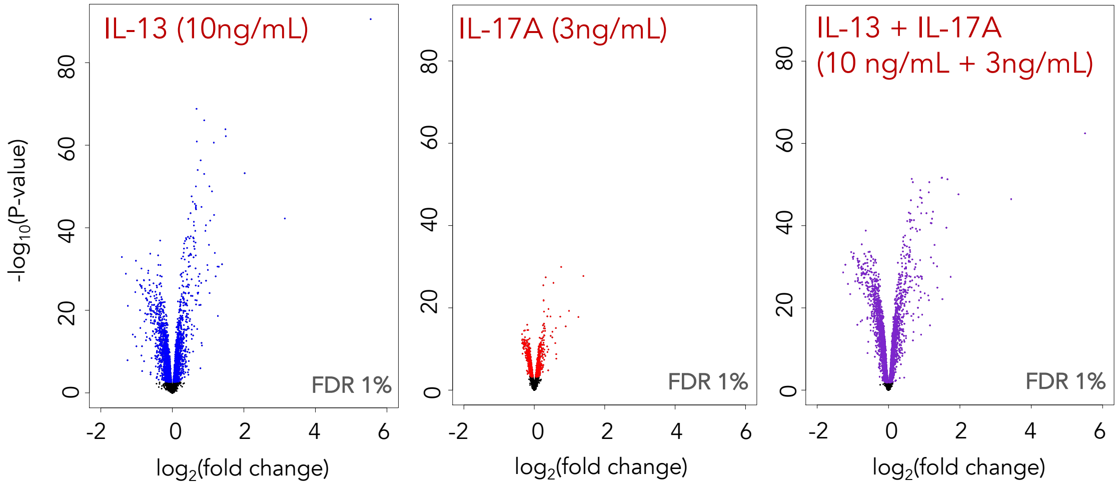

4105 response genes (2077 up, 2028 down) compared to vehicle

1059 response genes (469 up, 580 down) compared to vehicle

4519 response genes (2267 up, 2252 down) compared to vehicle
