# Supplementary material for: Cytokine-induced molecular responses in airway smooth muscle cells inform genome-wide association studies of asthma
Source: Genome Med. 2020 Jul 20;12:64. doi: 10.1186/s13073-020-00759-w (PMC7370514; doi:10.1186/s13073-020-00759-w)
Supplement: Supplementary file 5 — Additional file 5. Comparison of transcriptional responses to IL-17 from another study. Genes reported by Dragon et al. to be up-regulated in response to IL-17 exposure. [file 13073_2020_759_MOESM5_ESM.docx]

|  | *P* value reported in^1^ ^a^ | Fold change reported in^1^ | *P* value in this study (adj) ^b^ | Fold change in this study |
| --- | --- | --- | --- | --- |
| *NFKBIZ* | 0.000 | 4.48 | <2.2x10^-16^ | 18.99 |
| *ZC3H12A* | 0.000 | 1.98 | <2.2x10^-16^ | 20.78 |
| *NFKBIA* | 0.000 | 2.08 | Not detected |  |
| *RELB* | 0.000 | 1.40 | 0.01 | 3.53 |
| *BIRC3* | 0.001 | 1.55 | 0.61 | -1.18 |
| *CXCL1* | 0.003 | 2.55 | <2.2x10^-16^ | 12.15 |
| *ZC3H12C* | 0.003 | 1.21 | 4.98x10^-12^ | 9.97 |
| *TNFAIP3* | 0.005 | 1.72 | 0.52 | 1.36 |
| *MAP3K8* | 0.006 | 1.55 | <2.2x10^-16^ | 17.72 |
| *CXCL2* | 0.006 | 1.41 | <2.2x10^-16^ | 12.21 |
| *CASZ1* | 0.006 | 1.25 | 0.15 | 2.32 |
| *IER3* | 0.008 | 1.35 | 3.46x10^-11^ | 9.46 |

Additional File 5. Genes reported by Dragon et al. to be up-regulated in response to IL-17 exposure. Only genes with P<0.01 are shown.

^a^ ASMCs were exposed to 10 ng/mL IL-17 for two hours. N=6

^b^ ASMCs were exposed to 3 ng/mL IL-17 for 24 hours. N=67
