# Supplementary material for: Cytokine-induced molecular responses in airway smooth muscle cells inform genome-wide association studies of asthma
Source: Genome Med. 2020 Jul 20;12:64. doi: 10.1186/s13073-020-00759-w (PMC7370514; doi:10.1186/s13073-020-00759-w)

Additional File 7. Venn diagrams illustrating the numbers of DEGs (FDR<5%) in response to IL-13 (A), IL-17A (B), and IL-13+IL-17A (C) among ASMCs from individuals with (N=14) and without (N=53) asthma. D and E) Boxplots showing results of simulations to control for sample size between cases and controls. To generate these data, 100 sets of randomly sampled controls (N=14 and N=16 for expression and methylation data, respectively) were analyzed for differential gene expression or methylation against the same number of cases using limma. D) Median and range of DEGs among 100 random subsets of controls compared to the median number of DEGs in cases (red dot) for IL-13, IL-17A, and IL-13+IL-17A exposed ASMCs. E) Median and range of DMPs among controls compared to the median number of DMPs in cases (red dot).

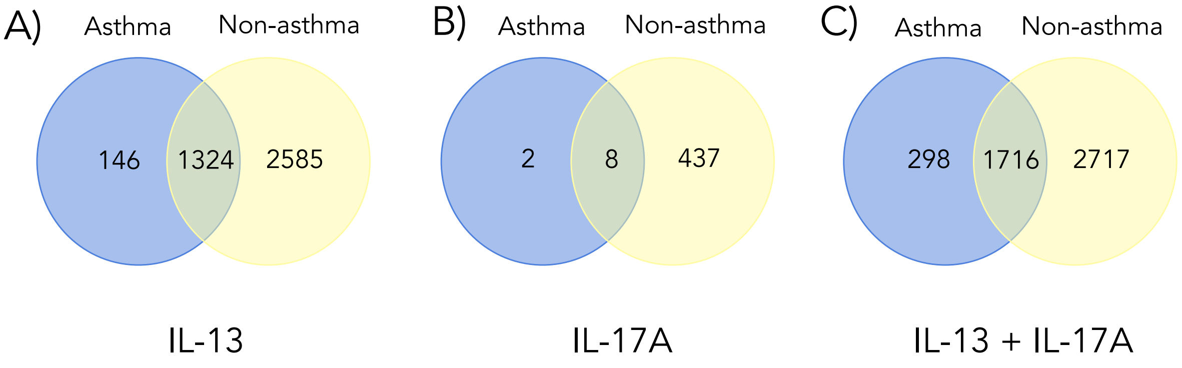

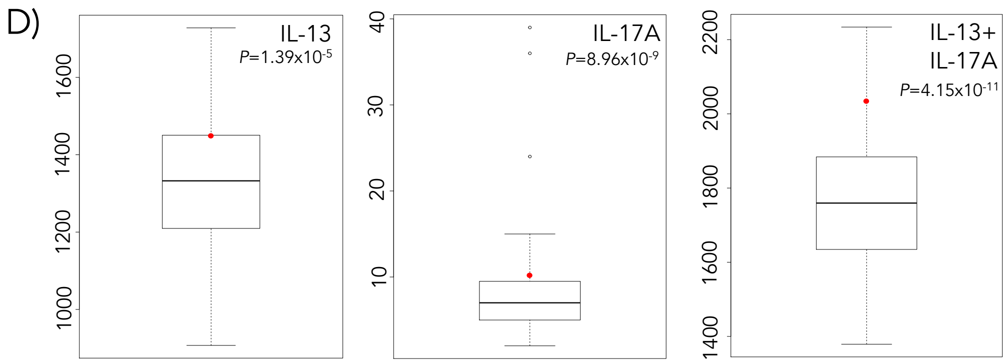

	Cases	Controls
	Mean (SD)	Mean (SD)
IL-13	1,413 (56.5)	1,332 (170.7)
IL-17A	10 (2.8)	7 (5.4)
IL-13+IL-17A	2,024 (61.4)	1,759 (177.6)

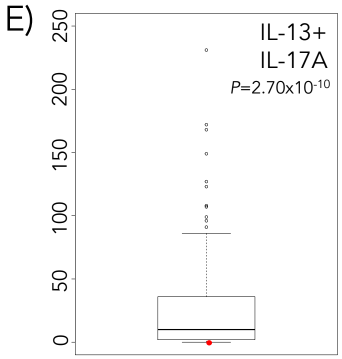

Supplement: Supplementary file 7 — Additional file 7. A comparison of differentially expressed genes among individuals with and without asthma. Information regarding numbers of DEGs (FDR<5%) in response to IL-13, IL-17A, and IL-13+IL-17A among ASMCs from individuals with (N=14) and without (N=53) asthma, plus boxplots showing results of simulations to control for sample size between cases and controls. [file 13073_2020_759_MOESM7_ESM.pdf]
