# Supplementary material for: Cytokine-induced molecular responses in airway smooth muscle cells inform genome-wide association studies of asthma
Source: Genome Med. 2020 Jul 20;12:64. doi: 10.1186/s13073-020-00759-w (PMC7370514; doi:10.1186/s13073-020-00759-w)
Supplement: Supplementary file 9 — Additional file 9. Volcano plots illustrating methylation responses to IL-13 and/or IL-17 in ASMCs. Methylation responses following 24 hours of exposure to IL-13, IL-17A, or IL-13+IL-17A compared to vehicle. [file 13073_2020_759_MOESM9_ESM.pdf]

Additional File 9. Methylation responses following 24 hours of exposure to IL-13, IL-17A, or IL-13+IL-17A compared to vehicle. A) Volcano plot of methylation in IL-13-exposed cells compared to vehicle. B) Volcano plot of methylation in IL-17A-exposed cells compared to vehicle. C) Volcano plot of methylation in IL-13 + IL-17A-exposed cells compared to vehicle. Colored dots represent transcripts DE at FDR<5%; dots in black are not significant in each panel. Log_2(fold change) is plotted along the X-axis and $-log_{10}$(*P*-value) is plotted on the Y-axis. D) Number of differentially methylated CpGs in ASMCs from individuals with and without asthma and in the full sample following cytokine exposure compared to vehicle. FDR=5%.

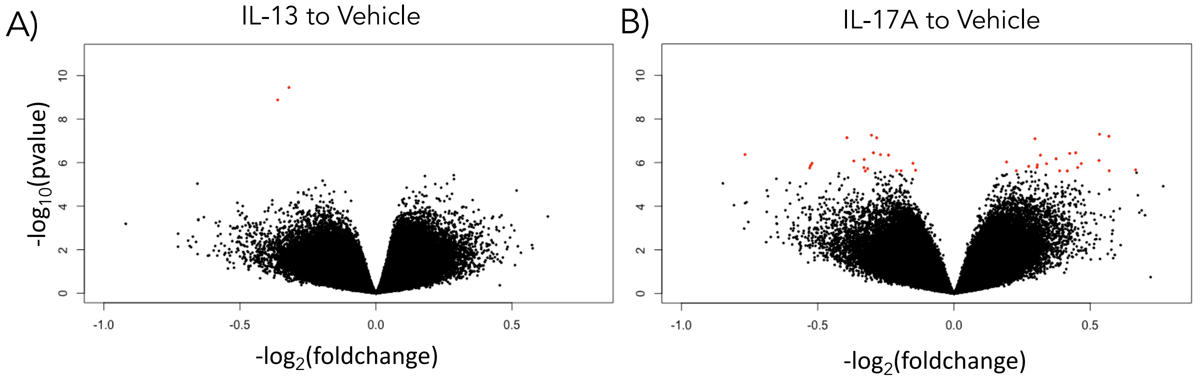

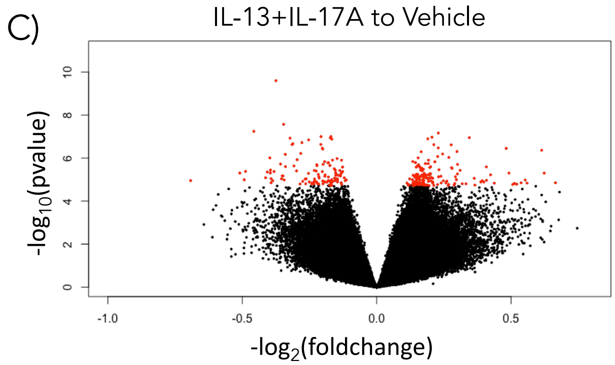

D)

	Asthma	Non-asthma	In full sample
IL-13	2	1	2
IL-17A	0	1	39
IL-13+IL-17A	0	260	167
