# Supplementary material for: Cytokine-induced molecular responses in airway smooth muscle cells inform genome-wide association studies of asthma
Source: Genome Med. 2020 Jul 20;12:64. doi: 10.1186/s13073-020-00759-w (PMC7370514; doi:10.1186/s13073-020-00759-w)

Additional File 10. Comparison of methylation levels at the 260 DMPs in controls following IL-13+IL-17 exposure to methylation levels among cases and controls at baseline (vehicle treated). The figure shows the absolute value of the change in methylation between the two groups: red circles = (exposed controls)-(baseline cases) and blue circles = (exposed controls)-(baseline controls). As the data suggests and the figure illustrates, methylation levels among cases at baseline (red) look more like the controls following IL-13+IL-17 stimulation.

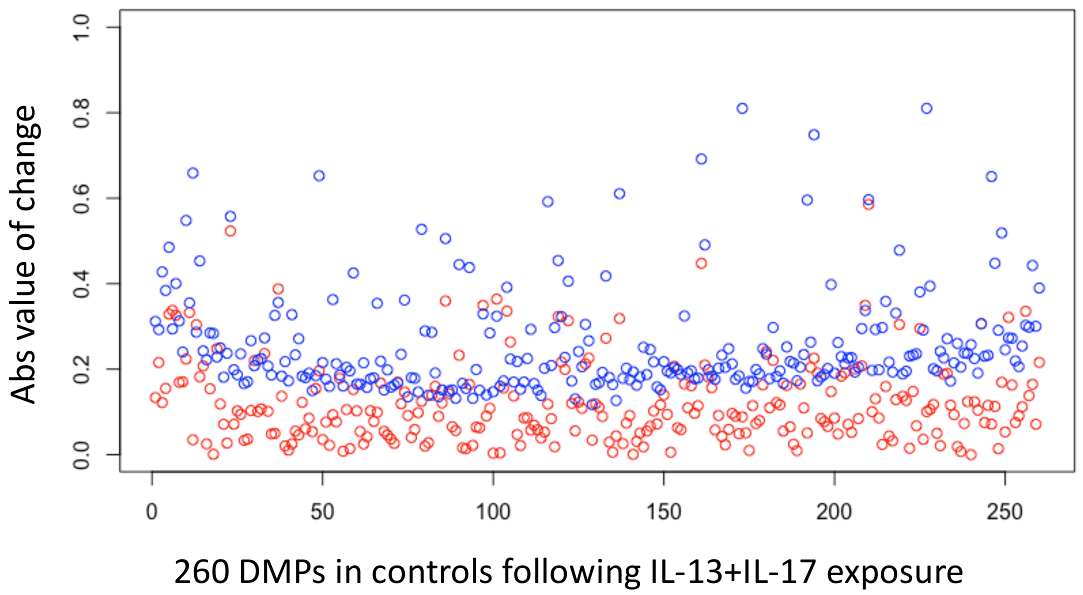

Supplement: Supplementary file 10 — Additional file 10. Methylation levels among cases at baseline (vehicle) look more like controls following IL-13+IL-17 stimulation. Comparison of methylation levels at the 260 DMPs in controls following IL-13+IL-17 exposure to methylation levels among cases and controls at baseline (vehicle treated). [file 13073_2020_759_MOESM10_ESM.pdf]
