# Supplementary material for: Cytokine-induced molecular responses in airway smooth muscle cells inform genome-wide association studies of asthma
Source: Genome Med. 2020 Jul 20;12:64. doi: 10.1186/s13073-020-00759-w (PMC7370514; doi:10.1186/s13073-020-00759-w)

Additional File 11. Comparison of contractile measurements in IL-13 and/or IL-17A-exposed cells. A) Comparison of RMS values between exposures. Measurements from cases appear as black dots and measurements from controls as red dots. B) Table showing mean and standard deviation of RMS values between exposures, separated by case-control status. None of the comparisons are significantly different between cases and controls. C) Density plot of correlations between transcript responses and contractile responses assessed for each exposure. Only correlations in the IL-17A-exposed ASMCs were enriched for small *P*-values.

A)

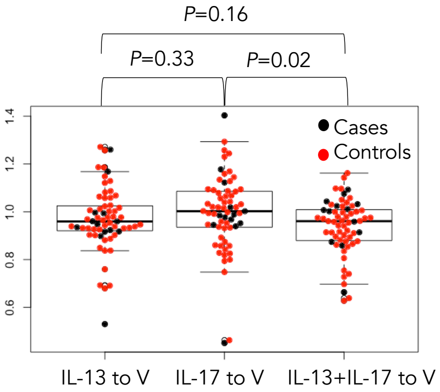

B)

	Vehicle[1]	IL-13[1]	IL-17[1]	IL-13+IL-17[1]
Cases	1.35 (0.26)	1.23 (0.19)	1.30 (0.28)	1.27 (0.23)
Controls	1.33 (0.20)	1.28 (0.18)	1.31 (0.19)	1.23 (0.17)
Total	1.33 (0.21)	1.27 (0.19)	1.31 (0.21)	1.23 (0.19)

[1] Difference between cases and controls *P*>0.44.

C)

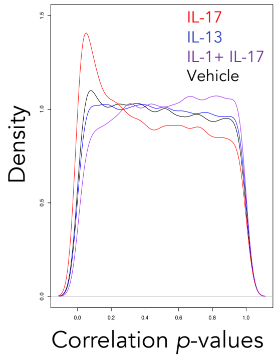

Supplement: Supplementary file 11 — Additional file 11. Contractile measurements do not significantly differ between cases and controls or among treatment groups. Comparison of contractile measurements in IL-13 and/or IL-17A-exposed cells. [file 13073_2020_759_MOESM11_ESM.pdf]
