# Supplementary material for: Cytokine-induced molecular responses in airway smooth muscle cells inform genome-wide association studies of asthma
Source: Genome Med. 2020 Jul 20;12:64. doi: 10.1186/s13073-020-00759-w (PMC7370514; doi:10.1186/s13073-020-00759-w)

Additional File 12. A) Q-Q plot of GWAS results for bronchical responsiveness index (BRI) in the Hutterites (N=964). B) Manhattan plot of GWAS results for bronchical responsiveness index (BRI) in the Hutterites (N=964).

A)

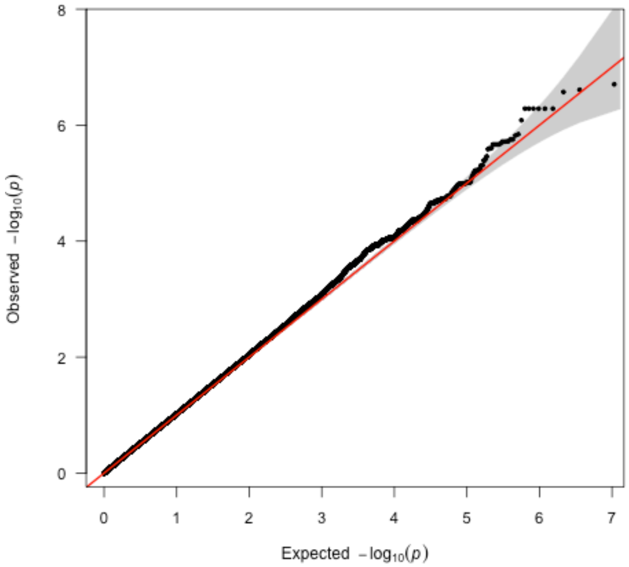

B)

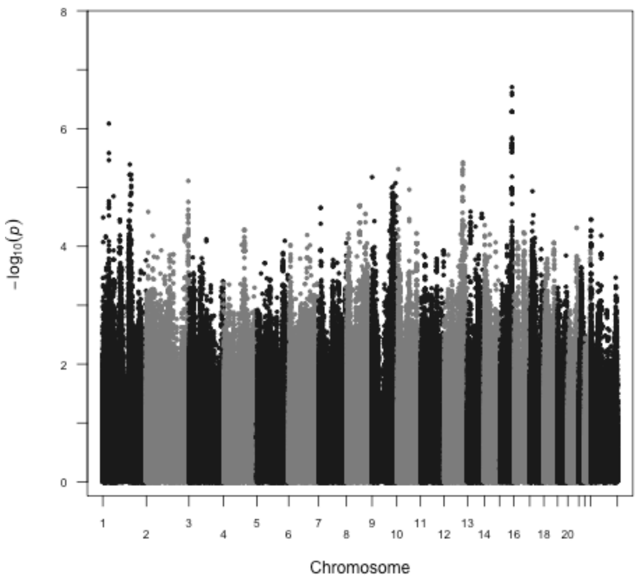

Supplement: Supplementary file 12 — Additional file 12. Summaries of GWAS for bronchial responsiveness in the Hutterites. Q-Q plot of GWAS results for bronchical responsiveness index (BRI) in the Hutterites (N=964) and Manhattan plot of GWAS results for bronchical responsiveness index (BRI) in the Hutterites (N=964). [file 13073_2020_759_MOESM12_ESM.pdf]
