# Supplementary material for: Cytokine-induced molecular responses in airway smooth muscle cells inform genome-wide association studies of asthma
Source: Genome Med. 2020 Jul 20;12:64. doi: 10.1186/s13073-020-00759-w (PMC7370514; doi:10.1186/s13073-020-00759-w)

Additional File 17. Venn diagram illustrating overlaps of molecular QTLs (e- and meQTLs; lfsr<0.05), BRI GWAS SNPs (P<0.01), and contractile response (co) QTLs (P<0.01).

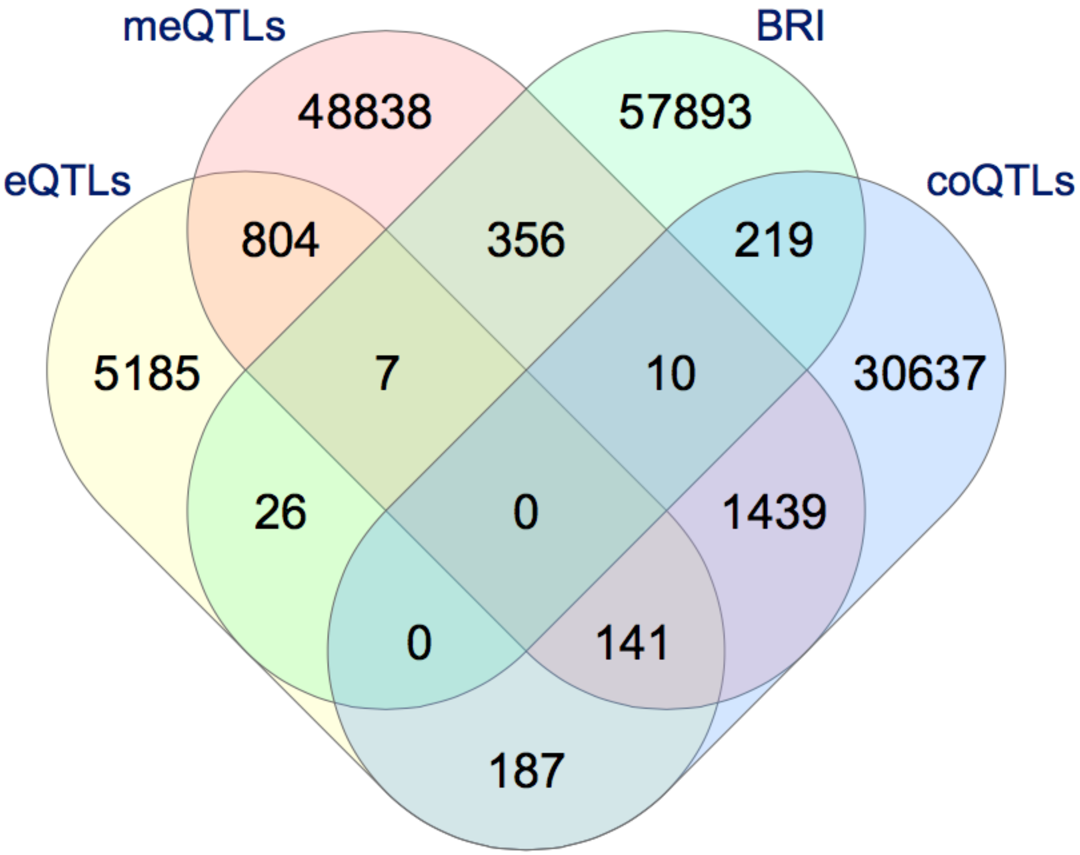

Supplement: Supplementary file 17 — Additional file 17. Overlaps of QTLs and GWAS SNPs identified in this study. Venn diagram illustrating overlaps of molecular QTLs (e- and meQTLs; lfsr<0.05), BRI GWAS SNPs (P<0.01), and contractile response (co) QTLs (P<0.01). [file 13073_2020_759_MOESM17_ESM.pdf]
