# Supplementary material for: Cytokine-induced molecular responses in airway smooth muscle cells inform genome-wide association studies of asthma
Source: Genome Med. 2020 Jul 20;12:64. doi: 10.1186/s13073-020-00759-w (PMC7370514; doi:10.1186/s13073-020-00759-w)
Supplement: Supplementary file 18 — Additional file 18. List of QTLs idenfitied in this study that are also BRI GWAS SNPs. eQTLs (lfsr<0.01), meQTLs (lfsr<0.05), and contractile response (co) QTLs (P<0.01) that are also BRI GWAS SNPs (P<0.01). [file 13073_2020_759_MOESM18_ESM.pdf]

Additional File 18. eQTLs (lfsr<0.01), meQTLs (lfsr<0.05), and contractile response (co)QTLs (*P*<0.01) that are also BRI GWAS SNPs (*P*<0.01).

Number	Source	Position	P value
1	eQTL	1:53073327	8.09E-08
2	eQTL	16:11343547	2.12E-05
3	eQTL	16:11345699	3.50E-05
4	eQTL	16:11350965	5.26E-05
5	eQTL	16:11351211	3.69E-05
6	eQTL	16:11355222	5.40E-05
7	eQTL	2:238968634	0.00143172
8	eQTL	20:21575866	0.00052856
9	eQTL	4:103569283	9.00E-05
10	eQTL	4:103578637	0.00013314
11	eQTL	4:103759189	2.38E-05
12	eQTL	4:103764993	8.05E-05
13	eQTL	4:103765916	8.59E-05
14	eQTL	4:103767339	8.12E-05
15	eQTL	4:103769234	1.73E-05
16	eQTL	4:103777392	5.51E-05
17	eQTL	4:103785857	3.91E-05
18	eQTL	4:103786588	3.81E-05
19	eQTL	4:103789810	3.42E-05
20	eQTL	4:103794536	2.95E-05
21	eQTL	4:103796908	2.72E-05
22	eQTL	4:103799532	2.45E-05
23	eQTL	5:76117065	1.88E-05
24	eQTL	5:76118333	1.05E-05
25	eQTL	5:76119049	1.60E-05
26	eQTL	5:76119105	1.20E-05
27	eQTL	5:76119365	5.31E-06
28	eQTL	5:76119712	1.35E-05
29	eQTL	5:76127135	4.06E-06
30	eQTL	5:76128334	9.72E-06
31	eQTL	7:90156945	0.0063208
32	eQTL	7:90210862	0.00936515
33	eQTL	7:90257651	0.01129679
1	meQTL	1:53187767	2.22E-16
2	meQTL	1:53187911	2.22E-16
3	meQTL	1:53189297	2.22E-16
4	meQTL	1:53195271	2.22E-16
5	meQTL	1:53195384	2.22E-16
6	meQTL	1:53195481	2.22E-16
7	meQTL	1:53195800	2.22E-16
8	meQTL	1:53196666	2.22E-16

9	meQTL	1:53196689	2.22E-16
10	meQTL	11:65638719	2.22E-16
11	meQTL	11:65641033	2.22E-16
12	meQTL	8:91678575	2.22E-16
13	meQTL	8:91680519	2.22E-16
14	meQTL	8:91680957	2.22E-16
15	meQTL	8:91681221	2.22E-16
16	meQTL	8:91681557	2.22E-16
17	meQTL	8:91682025	2.22E-16
18	meQTL	8:91682407	2.22E-16
19	meQTL	8:91682498	2.22E-16
20	meQTL	8:91683028	2.22E-16
21	meQTL	8:91684256	2.22E-16
22	meQTL	8:91685814	2.22E-16
23	meQTL	8:1809895	4.97E-18
24	meQTL	1:53193576	1.11E-16
25	meQTL	1:53193796	1.11E-16
26	meQTL	1:53195686	1.11E-16
27	meQTL	1:53195926	1.11E-16
28	meQTL	1:53196440	1.11E-16
29	meQTL	8:91677926	1.11E-16
30	meQTL	8:91678515	1.11E-16
31	meQTL	8:91678587	1.11E-16
32	meQTL	8:91678954	1.11E-16
33	meQTL	8:91679451	1.11E-16
34	meQTL	8:91684776	1.11E-16
35	meQTL	8:91685431	1.11E-16
36	meQTL	8:91676709	2.22E-16
37	meQTL	9:5610320	1.55E-15
38	meQTL	9:5612750	2.66E-15
39	meQTL	9:5612177	3.55E-15
40	meQTL	10:9804989	1.58E-11
41	meQTL	8:94754924	2.86E-11
42	meQTL	8:94754865	2.87E-11
43	meQTL	8:94753789	2.96E-11
44	meQTL	8:94745386	2.99E-11
45	meQTL	8:94750826	3.26E-11
46	meQTL	8:94752754	4.06E-11
47	meQTL	8:94749193	4.31E-11
48	meQTL	8:94749565	4.41E-11
49	meQTL	8:94750080	1.01E-10
50	meQTL	1:100182884	6.67E-10
51	meQTL	1:100185282	7.62E-10
52	meQTL	11:19466475	1.74E-09
53	meQTL	8:120166904	3.38E-09

54	meQTL	8:120166800	3.42E-09
55	meQTL	8:120163391	3.52E-09
56	meQTL	8:120163500	3.52E-09
57	meQTL	8:120164442	3.56E-09
58	meQTL	3:43794256	5.11E-09
59	meQTL	6:164242203	9.42E-09
60	meQTL	6:164242955	9.54E-09
61	meQTL	6:164243832	9.67E-09
62	meQTL	12:41219224	9.68E-09
63	meQTL	6:164246581	1.41E-08
64	meQTL	1:100047845	2.53E-08
65	meQTL	1:100049692	3.03E-08
66	meQTL	1:100050789	3.06E-08
67	meQTL	5:3339183	9.19E-08
68	meQTL	14:51230251	1.30E-07
69	meQTL	11:10398518	2.29E-07
70	meQTL	5:44816984	2.73E-07
71	meQTL	1:152362972	3.06E-07
72	meQTL	17:64579445	3.11E-07
73	meQTL	12:131608476	3.12E-07
74	meQTL	1:152363874	3.20E-07
75	meQTL	1:152365655	3.39E-07
76	meQTL	11:63058759	3.44E-07
77	meQTL	11:63058337	3.45E-07
78	meQTL	12:50531968	4.05E-07
79	meQTL	1:152360820	4.15E-07
80	meQTL	2:240178581	4.92E-07
81	meQTL	2:240178781	4.95E-07
82	meQTL	8:2879070	5.00E-07
83	meQTL	12:33145845	6.07E-07
84	meQTL	8:2881902	6.39E-07
85	meQTL	9:90709377	6.45E-07
86	meQTL	1:100048333	1.34E-06
87	meQTL	9:822594	1.83E-06
88	meQTL	1:97604184	1.89E-06
89	meQTL	3:194317010	2.04E-06
90	meQTL	1:97600910	2.16E-06
91	meQTL	4:120011540	2.16E-06
92	meQTL	1:97600310	2.27E-06
93	meQTL	9:90712434	2.49E-06
94	meQTL	9:90712312	2.66E-06
95	meQTL	9:90712331	2.67E-06
96	meQTL	12:33144023	2.81E-06
97	meQTL	12:33144084	2.81E-06
98	meQTL	12:33144337	2.81E-06

99	meQTL	12:33142560	2.83E-06
100	meQTL	12:33142937	2.83E-06
101	meQTL	9:90712552	3.06E-06
102	meQTL	5:76119365	4.66E-06
103	meQTL	8:8766536	5.60E-06
104	meQTL	8:8766635	5.68E-06
105	meQTL	2:45272197	5.81E-06
106	meQTL	3:194323620	7.65E-06
107	meQTL	12:1912526	8.58E-06
108	meQTL	12:50654894	9.95E-06
109	meQTL	8:8763983	1.05E-05
110	meQTL	11:65635559	1.12E-05
111	meQTL	1:152363201	1.59E-05
112	meQTL	1:152362727	1.65E-05
113	meQTL	15:77819778	1.92E-05
114	meQTL	16:85219630	2.00E-05
115	meQTL	7:11277857	2.09E-05
116	meQTL	8:114507204	2.27E-05
117	meQTL	15:77823013	2.30E-05
118	meQTL	8:114505160	2.36E-05
119	meQTL	8:114507145	2.38E-05
120	meQTL	20:61821519	2.41E-05
121	meQTL	7:11277413	2.55E-05
122	meQTL	16:85218567	2.62E-05
123	meQTL	20:61826127	2.98E-05
124	meQTL	20:61826132	2.98E-05
125	meQTL	12:32278032	3.11E-05
126	meQTL	13:97944331	3.65E-05
127	meQTL	8:120914270	3.82E-05
128	meQTL	8:120914432	3.82E-05
129	meQTL	8:120914527	3.82E-05
130	meQTL	8:120914908	3.82E-05
131	meQTL	8:120915232	3.83E-05
132	meQTL	8:120915862	3.83E-05
133	meQTL	8:120916394	3.83E-05
134	meQTL	8:120916555	3.84E-05
135	meQTL	8:120916651	3.84E-05
136	meQTL	17:32197482	4.22E-05
137	meQTL	17:32196551	4.99E-05
138	meQTL	17:32196268	5.10E-05
139	meQTL	17:32195732	5.16E-05
140	meQTL	17:32195167	5.23E-05
141	meQTL	17:32195714	5.31E-05
142	meQTL	8:114499211	6.32E-05
143	meQTL	5:76119712	6.39E-05

144	meQTL	16:8952113	6.40E-05
145	meQTL	5:76119049	6.50E-05
146	meQTL	8:114505777	6.57E-05
147	meQTL	16:85262290	6.63E-05
148	meQTL	5:76117065	6.74E-05
149	meQTL	6:121847256	6.81E-05
150	meQTL	4:120013271	6.99E-05
151	meQTL	8:114501219	7.41E-05
152	meQTL	7:157735442	7.72E-05
153	meQTL	20:61821068	8.74E-05
154	meQTL	5:76119105	8.81E-05
155	meQTL	11:93753048	9.47E-05
156	meQTL	11:93753037	9.48E-05
157	meQTL	11:93752680	9.50E-05
158	meQTL	11:93749872	9.63E-05
159	meQTL	11:93750050	9.63E-05
160	meQTL	15:74611781	0.00010924
161	meQTL	7:157735525	0.00011314
162	meQTL	8:11554261	0.00011571
163	meQTL	5:160229162	0.00011977
164	meQTL	5:160228932	0.00012013
165	meQTL	5:160228878	0.00012029
166	meQTL	6:121861658	0.00012787
167	meQTL	6:121860377	0.00012862
168	meQTL	6:121862617	0.00012937
169	meQTL	17:32198086	0.00013155
170	meQTL	5:76118333	0.00013474
171	meQTL	6:121862901	0.00013771
172	meQTL	13:103664563	0.00014809
173	meQTL	7:157735360	0.00015044
174	meQTL	5:160229610	0.00015259
175	meQTL	4:120011117	0.00015872
176	meQTL	16:85258569	0.00016002
177	meQTL	7:157742912	0.00016078
178	meQTL	16:8947128	0.00016212
179	meQTL	7:157742978	0.00016413
180	meQTL	5:160229021	0.00016684
181	meQTL	5:160227970	0.00017029
182	meQTL	12:114014227	0.00017472
183	meQTL	11:65630698	0.00017596
184	meQTL	7:157735216	0.00017696
185	meQTL	11:65623347	0.00017736
186	meQTL	18:60026931	0.00017747
187	meQTL	18:60027005	0.00017759
188	meQTL	11:65623519	0.00017801

189	meQTL	11:65621995	0.00017824
190	meQTL	11:65629934	0.00017945
191	meQTL	11:65630101	0.00017979
192	meQTL	16:85260802	0.00018004
193	meQTL	11:65631167	0.00018016
194	meQTL	5:160230008	0.00020975
195	meQTL	6:148882824	0.00022513
196	meQTL	4:103375303	0.0002454
197	meQTL	5:172094062	0.00025761
198	meQTL	15:48060331	0.00028099
199	meQTL	15:48061510	0.00028547
200	meQTL	8:120194297	0.00029299
201	meQTL	8:120193289	0.00030376
202	meQTL	8:120189693	0.00031489
203	meQTL	8:120189690	0.00031514
204	meQTL	8:120190284	0.00031739
205	meQTL	1:210002271	0.00031766
206	meQTL	7:157744142	0.00032117
207	meQTL	8:120189822	0.00032404
208	meQTL	8:120189729	0.00032442
209	meQTL	8:120189392	0.00032947
210	meQTL	8:120188880	0.00033382
211	meQTL	8:120188608	0.00033674
212	meQTL	8:120188617	0.00033981
213	meQTL	8:120187928	0.00034166
214	meQTL	8:120188188	0.00034626
215	meQTL	8:120187687	0.00035186
216	meQTL	8:11567140	0.00036077
217	meQTL	15:77820712	0.00040314
218	meQTL	15:77821331	0.00040501
219	meQTL	16:8949455	0.00042436
220	meQTL	7:2830243	0.00043287
221	meQTL	11:64412877	0.00066627
222	meQTL	11:67773897	0.00068801
223	meQTL	9:114703920	0.00069048
224	meQTL	7:157735966	0.00078443
225	meQTL	1:14448184	0.00080144
226	meQTL	11:64412086	0.00085162
227	meQTL	8:114451439	0.00086926
228	meQTL	1:18095997	0.0008736
229	meQTL	8:114448786	0.00088511
230	meQTL	1:14453185	0.00089888
231	meQTL	14:25518326	0.00108932
232	meQTL	7:157736304	0.00109375
233	meQTL	1:14449321	0.00122125

234	meQTL	7:157737139	0.00122951
235	meQTL	7:157736936	0.00125002
236	meQTL	2:9299699	0.00140137
237	meQTL	14:25515815	0.00142675
238	meQTL	8:102984352	0.0014351
239	meQTL	1:14447897	0.00154348
240	meQTL	7:157736235	0.00155561
241	meQTL	1:14448571	0.00156893
242	meQTL	1:14448388	0.00158264
243	meQTL	1:53073327	0.0017361
244	meQTL	16:55477585	0.00175692
245	meQTL	16:55477696	0.00179829
246	meQTL	3:196508551	0.00186682
247	meQTL	16:55475736	0.00187608
248	meQTL	16:55475668	0.00189671
249	meQTL	3:196507081	0.00206176
250	meQTL	14:25519106	0.00214072
251	meQTL	13:101316223	0.00248366
252	meQTL	15:74659609	0.00260113
253	meQTL	15:74661894	0.00262468
254	meQTL	3:196506153	0.00277882
255	meQTL	12:121168083	0.00303502
256	meQTL	8:8824837	0.00331557
257	meQTL	3:122609206	0.00339134
258	meQTL	2:101802078	0.00339447
259	meQTL	12:121170339	0.00341019
260	meQTL	3:42266346	0.00359962
261	meQTL	2:240294786	0.00362593
262	meQTL	2:240294969	0.00365737
263	meQTL	15:74662811	0.00451017
264	meQTL	2:103297704	0.00465448
265	meQTL	5:153582454	0.00570937
266	meQTL	3:27867461	0.00571931
267	meQTL	17:15414354	0.00642809
268	meQTL	20:47939664	0.00678305
269	meQTL	5:172093821	0.00764608
270	meQTL	15:59697546	0.00765787
271	meQTL	11:20899163	0.00770956
272	meQTL	15:59697236	0.00780102
273	meQTL	5:172092620	0.00812834
274	meQTL	11:63039972	0.00820448
275	meQTL	11:65631881	0.0091904
276	meQTL	11:65632262	0.00933662
277	meQTL	5:9854563	0.0094975
278	meQTL	16:55479687	0.00957827

279	meQTL	16:55481549	0.00978943
280	meQTL	16:55482198	0.00986373
281	meQTL	5:9853378	0.00986841
282	meQTL	16:55482117	0.00988274
283	meQTL	16:55478822	0.00991242
284	meQTL	16:55478046	0.00992066
285	meQTL	16:55478701	0.00992219
286	meQTL	16:55477451	0.00995967
287	meQTL	16:55480809	0.00998588
288	meQTL	16:55483037	0.01001989
289	meQTL	16:55477447	0.01005513
290	meQTL	16:55481269	0.01006834
291	meQTL	8:94242523	0.01009192
292	meQTL	16:55475437	0.01013791
293	meQTL	9:116935764	0.01024341
294	meQTL	16:55476494	0.01025544
295	meQTL	9:116935696	0.01026014
296	meQTL	16:55476449	0.01027733
297	meQTL	1:157084039	0.0107677
298	meQTL	5:9851399	0.01105215
299	meQTL	2:9285990	0.0115177
300	meQTL	16:55483244	0.0117342
301	meQTL	16:55479400	0.01186086
302	meQTL	16:55478444	0.01188712
303	meQTL	16:55479576	0.01231748
304	meQTL	16:55479525	0.01236153
305	meQTL	16:55478556	0.01240523
306	meQTL	16:55478526	0.01240966
307	meQTL	2:10361023	0.0125052
308	meQTL	16:55477159	0.01255584
309	meQTL	16:55477008	0.01261012
310	meQTL	16:55476322	0.01262558
311	meQTL	16:55476343	0.01272386
312	meQTL	16:55475874	0.01273993
313	meQTL	16:55476052	0.01278148
314	meQTL	16:55475129	0.01284986
315	meQTL	16:55475352	0.01288154
316	meQTL	16:55482873	0.01359253
317	meQTL	14:66254578	0.01373768
318	meQTL	9:119616042	0.01400218
319	meQTL	2:9285525	0.01438464
320	meQTL	10:14535113	0.01459751
321	meQTL	20:50423564	0.0147066
322	meQTL	2:9284616	0.01519562
323	meQTL	10:112586096	0.0153454

324	meQTL	8:94715291	0.01535933
325	meQTL	14:66255781	0.01547221
326	meQTL	11:64377336	0.01551171
327	meQTL	9:116935688	0.01573551
328	meQTL	2:9285206	0.01593781
329	meQTL	16:69163143	0.01666844
330	meQTL	11:64372302	0.01668672
331	meQTL	11:65621293	0.01899393
332	meQTL	11:65621057	0.0190182
333	meQTL	14:56711146	0.0194257
334	meQTL	12:120393756	0.02078294
335	meQTL	2:10288547	0.02109612
336	meQTL	14:66253437	0.02142277
337	meQTL	2:10359271	0.02283771
338	meQTL	15:59701847	0.02283888
339	meQTL	1:161043764	0.02378405
340	meQTL	14:25425252	0.02424066
341	meQTL	15:59701640	0.02529355
342	meQTL	8:107833222	0.02565955
343	meQTL	1:161043735	0.03004231
344	meQTL	8:107835922	0.0307742
345	meQTL	16:55528127	0.03125284
346	meQTL	16:55527986	0.03129493
347	meQTL	8:107836172	0.03138101
348	meQTL	9:100528320	0.03148157
349	meQTL	8:107836362	0.03166953
350	meQTL	12:50583872	0.03185783
351	meQTL	8:107836447	0.03203826
352	meQTL	8:107836730	0.03225098
353	meQTL	8:107836800	0.03249408
354	meQTL	10:23659587	0.03343644
355	meQTL	14:25423714	0.03361192
356	meQTL	16:55527026	0.03379528
357	meQTL	8:107837750	0.03421377
358	meQTL	16:55527113	0.03439357
359	meQTL	14:56711824	0.03589328
360	meQTL	10:23660531	0.03668929
361	meQTL	10:6504126	0.03670823
362	meQTL	10:23661291	0.03716188
363	meQTL	16:81870022	0.03819542
364	meQTL	8:107840839	0.03829959
365	meQTL	8:107839128	0.03883914
366	meQTL	8:107839322	0.03942668
367	meQTL	8:107839337	0.03942802
368	meQTL	8:107841205	0.03996542

369	meQTL	20:18265525	0.04302224
370	meQTL	14:22248839	0.04343635
371	meQTL	14:22248348	0.04437434
372	meQTL	20:50423643	0.0466798
373	meQTL	15:69454261	0.04746009
1	coQTL	1:95057293	0.00937089
2	coQTL	1:107002447	0.00054533
3	coQTL	1:107004365	0.00054533
4	coQTL	1:107009801	0.001705
5	coQTL	1:157381033	0.00895797
6	coQTL	2:10671555	0.00032107
7	coQTL	2:39791925	0.00882209
8	coQTL	2:40947022	0.00356295
9	coQTL	2:40949168	0.00295292
10	coQTL	2:40950118	0.00356295
11	coQTL	2:42136996	0.00421699
12	coQTL	2:42137215	0.00106134
13	coQTL	2:42137293	0.00073741
14	coQTL	2:43839090	0.00734334
15	coQTL	2:43839896	0.00734334
16	coQTL	2:43840048	0.00734334
17	coQTL	2:43841119	0.00734334
18	coQTL	2:43841269	0.00704516
19	coQTL	2:82679503	0.00121157
20	coQTL	2:82688980	0.00190449
21	coQTL	2:103559294	0.0076086
22	coQTL	2:133629564	0.00054588
23	coQTL	2:239417840	0.00377577
24	coQTL	3:14242148	0.00809908
25	coQTL	3:24680131	0.00057624
26	coQTL	3:24685069	0.00057624
27	coQTL	3:26124633	0.00196199
28	coQTL	3:77377533	0.00542131
29	coQTL	3:77377731	0.00542131
30	coQTL	3:77378207	0.0054261
31	coQTL	3:77382285	0.00991803
32	coQTL	3:77384295	0.00991803
33	coQTL	3:115981443	0.00491336
34	coQTL	3:115982415	0.00928987
35	coQTL	3:115983248	0.00928987
36	coQTL	4:104293598	0.00673255
37	coQTL	4:183383425	0.00583007
38	coQTL	5:9844926	0.00974845
39	coQTL	5:9851399	0.0095592
40	coQTL	5:9853378	0.00904782

41	coQTL	5:9854563	0.00904782
42	coQTL	5:31839299	0.00937666
43	coQTL	5:58676173	0.00762617
44	coQTL	5:172145086	0.00982512
45	coQTL	5:172146166	0.00982512
46	coQTL	5:172147182	0.00962384
47	coQTL	5:172148182	0.00962384
48	coQTL	5:172148934	0.00962384
49	coQTL	6:164146179	0.00389594
50	coQTL	8:1600669	0.00498536
51	coQTL	8:1603043	0.00412249
52	coQTL	8:13564225	0.00583752
53	coQTL	8:72618673	0.00458044
54	coQTL	8:72619458	0.00458044
55	coQTL	8:72619613	0.00458044
56	coQTL	8:72620818	0.00421761
57	coQTL	8:76476396	0.00920932
58	coQTL	8:76476457	0.00920932
59	coQTL	8:76476670	0.00920932
60	coQTL	8:76477368	0.00920932
61	coQTL	8:102981279	0.00734969
62	coQTL	8:122002595	0.0090656
63	coQTL	8:122002754	0.0090656
64	coQTL	9:1632203	0.00843994
65	coQTL	9:5610320	0.00931348
66	coQTL	9:5611912	0.00962145
67	coQTL	9:5612177	0.00464447
68	coQTL	9:5612750	0.00674231
69	coQTL	9:20781979	0.00868207
70	coQTL	9:82457250	0.00571477
71	coQTL	9:117855507	0.0024783
72	coQTL	10:9192570	2.31E-05
73	coQTL	10:9193193	2.18E-05
74	coQTL	10:9193901	2.18E-05
75	coQTL	10:9193998	2.18E-05
76	coQTL	10:9194006	2.18E-05
77	coQTL	10:9196330	2.32E-05
78	coQTL	10:9197666	2.32E-05
79	coQTL	10:9200102	0.00025048
80	coQTL	10:9203792	0.00024103
81	coQTL	10:9205270	0.00024103
82	coQTL	10:9207621	2.19E-05
83	coQTL	10:9207877	2.19E-05
84	coQTL	10:9207928	2.19E-05
85	coQTL	10:9826151	0.00738161

86	coQTL	10:14501236	0.00726919
87	coQTL	10:49942417	0.00581547
88	coQTL	10:52006233	0.00788291
89	coQTL	10:52006252	0.00632516
90	coQTL	10:52006502	0.00788291
91	coQTL	10:52006528	0.00632516
92	coQTL	10:52006649	0.00788291
93	coQTL	10:52006741	0.00788291
94	coQTL	10:52006771	0.00788291
95	coQTL	10:52007179	0.00788291
96	coQTL	10:52007281	0.00788291
97	coQTL	10:52007347	0.00788291
98	coQTL	10:52007626	0.00632516
99	coQTL	10:52008748	0.0025629
100	coQTL	10:52009061	0.00788291
101	coQTL	10:61744247	0.00132772
102	coQTL	10:61745158	0.00350281
103	coQTL	10:61745771	0.00350281
104	coQTL	10:85242245	0.00986481
105	coQTL	10:85258657	0.00756431
106	coQTL	10:85258710	0.00756431
107	coQTL	11:8948058	0.00670342
108	coQTL	11:19435430	0.0068028
109	coQTL	11:19435457	0.0068028
110	coQTL	11:19435829	0.00777041
111	coQTL	11:19439399	0.00777041
112	coQTL	11:19439463	0.00777041
113	coQTL	11:19439588	0.00777041
114	coQTL	11:19439595	0.00777041
115	coQTL	11:19440354	0.00777041
116	coQTL	11:19440455	0.00777041
117	coQTL	11:19440493	0.00752438
118	coQTL	11:19440567	0.00777041
119	coQTL	11:19441522	0.00752438
120	coQTL	11:19441717	0.00777041
121	coQTL	11:19441858	0.00777041
122	coQTL	11:19442926	0.00752438
123	coQTL	11:19466475	0.00720895
124	coQTL	11:65458310	0.00494324
125	coQTL	11:65464000	0.00494324
126	coQTL	12:83422599	0.00067548
127	coQTL	12:83423340	0.00057492
128	coQTL	12:83424175	0.00096374
129	coQTL	12:84143176	0.00622051
130	coQTL	12:92174205	0.00210524

131	coQTL	12:96435239	0.00448924
132	coQTL	12:96437926	0.00595761
133	coQTL	12:103542414	0.0006334
134	coQTL	12:104182968	0.00715356
135	coQTL	12:105689161	0.00610841
136	coQTL	12:105689315	0.00610841
137	coQTL	12:105689407	0.00610841
138	coQTL	12:105689523	0.00610841
139	coQTL	12:105689566	0.00844684
140	coQTL	12:105690017	0.00176446
141	coQTL	12:105690259	0.00610841
142	coQTL	12:105691466	0.00610841
143	coQTL	12:105694513	0.00610841
144	coQTL	12:105694812	0.00610841
145	coQTL	12:105695312	0.00585921
146	coQTL	12:105695459	0.00610841
147	coQTL	12:105695541	0.00610841
148	coQTL	12:105695861	0.00610841
149	coQTL	12:107415073	0.00569431
150	coQTL	12:107417182	0.00569431
151	coQTL	12:107423275	0.00569431
152	coQTL	12:107440145	0.00569431
153	coQTL	12:107440824	0.00569431
154	coQTL	12:107443546	0.00569431
155	coQTL	12:107445137	0.00569431
156	coQTL	12:107445881	0.00569431
157	coQTL	12:107454152	0.00569431
158	coQTL	12:107459096	0.00569431
159	coQTL	12:107472206	0.00569431
160	coQTL	12:107477523	0.00472731
161	coQTL	12:107483888	0.00472731
162	coQTL	12:107487069	0.00639625
163	coQTL	12:107488186	0.00472731
164	coQTL	12:107489155	0.00472731
165	coQTL	12:107491592	0.00639625
166	coQTL	12:107497664	0.00581391
167	coQTL	12:107499824	0.00472731
168	coQTL	12:107503125	0.00472731
169	coQTL	12:107505640	0.00639625
170	coQTL	12:107505669	0.00472731
171	coQTL	12:107506174	0.00472731
172	coQTL	12:107507728	0.00472731
173	coQTL	13:97522994	0.0066547
174	coQTL	13:97525122	0.0066547
175	coQTL	13:97525422	0.0066547

176	coQTL	13:97525809	0.0066547
177	coQTL	13:97526119	0.0066547
178	coQTL	13:97528123	0.00736826
179	coQTL	15:58740094	0.00367498
180	coQTL	15:84441823	0.00764143
181	coQTL	15:84452957	0.00764143
182	coQTL	15:84453788	0.00764143
183	coQTL	15:84453883	0.00764143
184	coQTL	15:91151276	0.00074957
185	coQTL	15:91152911	0.00063644
186	coQTL	15:91180705	0.00517847
187	coQTL	15:91186461	0.00203955
188	coQTL	16:8947128	0.00846907
189	coQTL	16:8949455	0.0054626
190	coQTL	16:16037261	0.00850095
191	coQTL	16:61041750	0.00356953
192	coQTL	16:61041758	0.00356953
193	coQTL	16:61042482	0.00356953
194	coQTL	16:61042822	0.00356953
195	coQTL	16:61042959	0.00356953
196	coQTL	16:61046387	0.00356953
197	coQTL	16:61046749	0.00356953
198	coQTL	16:69131281	0.00461822
199	coQTL	17:32472860	0.0099506
200	coQTL	18:18559675	0.00374803
201	coQTL	18:18602977	0.00845554
202	coQTL	18:19100854	0.00023254
203	coQTL	18:59321737	0.00017108
204	coQTL	20:47854020	0.00207179
205	coQTL	20:47857067	0.00224721
206	coQTL	20:47866411	0.00224721
207	coQTL	20:47889958	0.002077
208	coQTL	20:47900164	0.0021668
209	coQTL	20:47903826	0.00225637
210	coQTL	20:47913578	0.00225637
211	coQTL	20:50423643	0.00103109
212	coQTL	20:50446692	0.00165171
213	coQTL	20:50449214	0.00036901
214	coQTL	20:50449250	0.00018053
215	coQTL	20:50449781	0.00018053
216	coQTL	20:50450541	0.00039486
217	coQTL	20:50450560	0.00018053
218	coQTL	20:50451294	0.00019926
219	coQTL	20:50451967	0.00033897
220	coQTL	20:50452198	0.00019926

221	coQTL	20:50455228	0.00219286
222	coQTL	20:50455735	0.00014805
223	coQTL	20:50455767	0.00182103
224	coQTL	20:50455860	0.00024283
225	coQTL	20:50456014	0.00024283
226	coQTL	20:50456180	0.00024283
227	coQTL	20:50456237	0.00024283
228	coQTL	21:30309552	0.00399409
229	coQTL	21:30312630	0.0029069
